# Variable characteristics overlooked in human K-562 leukemia cell lines with a common signature

**DOI:** 10.1038/s41598-024-60271-8

**Published:** 2024-04-26

**Authors:** Fumio Kasai, Kumiko Mizukoshi, Yukio Nakamura

**Affiliations:** RIKEN Cell Bank, Cell Engineering Division, RIKEN BioResource Research Center, Tsukuba, Japan

**Keywords:** Cell line authentication, Cell name, Cell subline, In vitro cellular model, Reference data, Biological models, Cancer models

## Abstract

K-562 is a well-known in vitro cellular model that represents human leukemia cell lines. Although the K-562 cells have been extensively characterized, there are inconsistencies in the data across publications, showing the presence of multiple K-562 cell lines. This suggests that analyzing a single K-562 cell line is insufficient to provide reliable reference data. In this study, we compared three K-562 cell lines with different IDs (RCB0027, RCB1635, and RCB1897) to investigate the fundamental characteristics of K-562 cells. Amplifications of the *BCR-ABL1* fusion gene and at 13q31 were detected in all three cell lines, whereas each genome exhibited distinctive features of sequence variants and loss of heterozygosity. This implies that each K-562 cell line can be characterized by common and unique features through a comparison of multiple K-562 cell lines. Variations in transcriptome profiles and hemoglobin synthesis were also observed among the three cell lines, indicating that they should be considered sublines that have diverged from the common ancestral K-562 despite no changes from the original cell name. This leads to unintentional differences in genotypes and/or phenotypes among cell lines that share the same name. These data show that characterizing a single K-562 cell line does not necessarily provide data that are applicable to other K-562 cells. In this context, it is essential to modify cell names in accordance with changes in characteristics during cell culture. Furthermore, our data could serve as a reference for evaluating other K-562 sublines, facilitating the discovery of new K-562 sublines with distinct characteristics. This approach results in the accumulation of K-562 sublines with diverged characteristics and expands the options available, which may help in selecting the most suitable K-562 subline for each experiment.

## Introduction

K-562 is the first cell line reported in 1975 from a patient with chronic myelogenous leukemia (CML) in blast crisis^1^. As the phenotype was erythroleukemia, K-562 cells could be induced to differentiate into mature erythroid cells that express hemoglobin^2,3^. It is one of the frequently used cancer cell lines in publications and continues to serve as a representative human leukemia cell line.

Typical CML is characterized by the presence of the Philadelphia chromosome^4^. This was initially identified in the K-562 karyotype^5^, which subsequently led to the discovery of the *BCR-ABL1* fusion gene^6^. K-562 is listed as an in vitro model for Philadelphia chromosome-positive leukemia^7^. However, the absence of the Philadelphia chromosome had been noted by chromosome analysis^8–10^. Although the K-562 karyotype initially consisted of 46 chromosomes^5^, it became near triploid after being transplanted into nude mice^5^. This suggests that a ploidy change occurred during the early stages of the K-562 cell lineage, raising concerns about their suitability as a representative in vitro model for CML. The true story of the K-562 cell line has been missed, despite its widespread use.

The Cancer Cell Line Encyclopedia (CCLE) and the Catalogue of Somatic Mutations in Cancer (COSMIC) play important roles as databases of cancer cell lines. Although a detailed analysis was performed on the K-562 cell line stored in the Stanford ENCODE Product Center^11^, the datasets may only be applicable to the specific K-562 cell line used in the ENCODE study. Databases or publications provide comprehensive datasets for K-562, which are generally based on the analysis of a single K-562 subline. Whereas, differences in cancer cell lines across laboratories have been extensively documented for widely used cell lines, such as MCF7^12^ and HeLa^13^. It is expected that K-562 cells could undergo further divergence through in vitro cell culture, resulting in several sublines with distinct characteristics. Data on K-562 cell lines have been accumulated; however, it seems that reliable reference data for K-562, accompanied by publicly available cellular materials, have not been established.

Misidentification or cross-contamination between human cell lines can be excluded through STR polymorphism analysis, which confirms the identity of their origins^14–16^. Although differences in STR profiles between cell lines indicate genetic drift, samples with more than an 80% match have been accepted as long as the cell name is identical, despite changes that occurred during cell culture^16^. Cell line authentication by STR analysis is mandatory for submitting manuscripts to major journals as it helps prevent the publication of misidentified cell lines^17^. However, it is worth noting that differences among sublines have not been measured in the conventional cell line authentication.

The K-562 cell line has been registered in multiple cell repositories, including ATCC, DSMZ, ECACC, and JCRB (Table 1). The RIKEN Cell Bank also maintains K-562 with three different cell IDs, although they all share the same name. However, a detailed analysis to determine possible differences between the three lines has not been performed. In this study, the three K-562 cell lines have been assessed based on their genome profiles, which are compared with their transcriptome signatures.

**Table 1:** Availability of K-562 cell line in major public cell banks.

Cellosaurus	Cell bank	Reference/cell ID no	Culture medium
CVCL_0004	Establishment	Lozzio1975	MEM + 15% FBS + NEAA
	RIKEN	RCB0027	HamF12 + 10% FBS
		RCB1635	MEM + 15% FBS + 0.1 mM NEAA
		RCB1897	RPMI1640 + 10% FBS
	JCRB	JCRB0019	RPMI1640 + 10% FBS
	ATCC	CCL-243	IMDM + 10% FBS
	DSMZ	ACC-10	RPMI1640 + 10% FBS
	ECACC	89121407	RPMI1640 + 10% FBS

## Results

### DNA profiling using 24 STR markers

Electropherograms from the STR analysis are shown in Fig. S1 and the corresponding profiles are listed in Table S2. The profiles show differences in 10 markers among the three K-562 sublines. The detection of a single allelic pattern at seven loci (D2S1338, D3S1358, D10S1248, vWA, D12S391, D13S317 and D22S1045) corresponds to loss of heterozygosity (LOH) regions that are common to the three sublines. Additional single allelic patterns at D2S441, D16S539 and D19S433 are specific to RCB1897 and could also be caused by LOH occurring only in the RCB1897 lineage. Although D12S391 and D21S11 in RCB0027 are located within LOH regions, their allelic patterns exhibit heterozygosity because of the formation of a new variant allele. The detection of three allelic patterns at three loci (D1S1656, D5S818 and D16S539) is specific to RCB1635. These changes in repeat numbers could be caused by the insertion or deletion of a repeating unit in one of the alleles, leading to the emergence of an additional variant allele. Differences in STR patterns are reflected by genomic changes that occur in each cell lineage, enabling the distinction of the three K-562 sublines.

### Cytogenetic features

Based on a count of 50 metaphases from RCB0027 and RCB1635, their modal chromosome numbers were 67 and 65, respectively (Fig. S2). Both can be classified as hypo-triploid. An early study reported variability among K-562 cell lines^18^, which is reflected in differences in M-FISH karyotypes between publications^9,10,19^. Chromosome numbers vary between cells, indicating a heterogeneous cell population.

### Whole genome profiles

**Figure 1:**
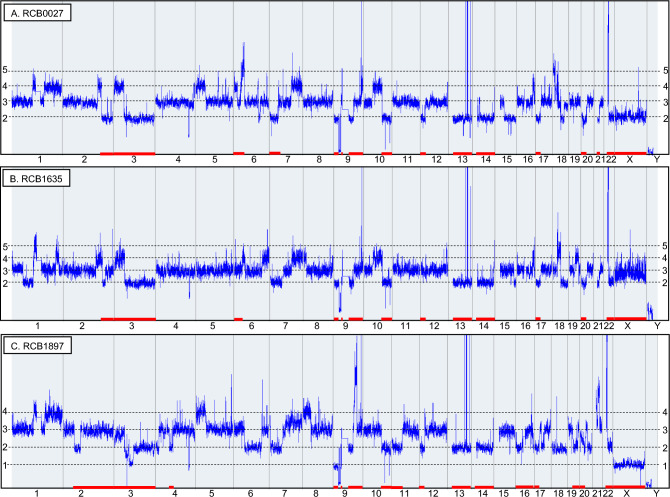
Copy number profiles compared among three K-562 sublines based on DNA microarray analysis. The horizontal and vertical axes represent chromosomes and DNA copy numbers, respectively. The red bars above the chromosome numbers indicate the loss of heterozygosity, which is listed in Table S3. The regions of LOH commonly observed in the three sublines exceeds 900 Mb, with additional changes detected in each lineage. Amplifications in chromosomes 9, 13, and 22 (Fig. S4D–F), and deletions in chromosome 9 are identical across all three profiles (Fig. S4C). RCB0027 (**A**) exhibits complex rearrangements on chromosome 18, unlike the other two sublines (Fig. S4A). RCB1635 (**B**) can be characterized by having 2 or 3 mosaic copies of the X chromosome, which is different from RCB0027 that has two copies and RCB1897 that has only one copy. RCB1897 (**C**) exhibits a high level of LOH, spanning a total of 1193.7 Mb in total. Whole profiles, including allele differences based on SNP data, are presented in Fig. S3.

The DNA copy number profiles of the three K-562 genomes are compared in Fig. 1, while allelic differences in each cell line are depicted in Fig. S3. Copy number alterations are listed in Table S3. Gains and/or losses are detected in every chromosome, indicating that none of the chromosomes are in a normal state showing a pair of heterozygous compositions. The major patterns of allelic differences observed in the three K-562 sublines consist of four bands. These bands correspond to heterozygous three copies, which result from the duplication of one of the alleles. This is reflected in the karyotypes of RCB0027 and RCB1635, which show hypo-triploid. RCB1897 is similar to the other two sublines, suggesting that its karyotype is also hypo-triploid.

The DNA copy number profile of chromosome 18 varies significantly among the three sublines (Fig. S4A). Except for the cryptic gain commonly observed at 18q11.2, which includes a partial *CHST9* gene, RCB1897 appears to be normal. However, RCB0027 exhibits more complex changes than RCB1635. Losses in the *FHIT* gene at 4p14.2 occurred in introns 5–8, with variations observed among the sublines (Fig. S4B). Deletions at 9p21, which include *CDKN2A/B*, are observed in all three sublines (Fig. S4C). Although the X chromosome of RCB0027 appears to have a normal copy number, the allelic composition is homozygous in all three sublines. The copy number of the X chromosome is 2 in RCB0027, 2/3 in RCB1635, and 1 in RCB1897. This variation in copy number could serve as a marker to distinguish the K-562 sublines at the chromosome level.

Amplification at 9q34 extends to 450 kb between intron 1 of *ABL1* and intron 29 of *NUP214* (Fig. S4D). The gain at 22q11.2 spans 4.6 Mb between *DGCR2* and *BCR*, which includes the amplification of 900 kb between *ZNF280B* and intron 14 of *BCR* (Fig. S4E). Another gain is observed at 22q11.1, involving *CCT8L2*, *TPTEP1* and *XKR3* (Fig. S4E). These copy number changes correspond to the amplification of the *BCR-ABL1* fusion gene^20,21^, leading to the absence of the Philadelphia chromosome in M-FISH or G-banding karyotypes^8–10,19^. Amplification at 13q31 is divided into three regions, comprising 2 Mb, 400 kb and 150 kb (Fig. S4F). It is noted that the amplification involves *MIR17HG* and the breakpoint is located within *GPC5*, resulting in partial amplification of *GPC5*. Amplification at 13q31-32 has been reported in lymphoma, indicating that *GPC5*^22^ and *C13orf25*^23^ correspond to *MIR17HG* as the targets. The patterns of these amplifications are identical across the three sublines, indicating a shared characteristic of K-562 cell lines. A focal gain spanning 2.8 Mb, including *MYC*, *PVT1*, and *ASAP1*, is uniquely detected in RCB1635 (Fig. S4G).

LOH are extensively observed in the K-562 genome, as illustrated by the copy number profiles shown in each line (Fig. 1). LOHs larger than 4 Mb are listed in Table S4, providing the total size of these regions. Among the three sublines, RCB1635 has the smallest total size of the total LOH, which is also present in the other two sublines. RCB1897 has five additional LOHs, comprising a total of 1193.7 Mb, indicating a highly altered genome. The LOH at 6p25.3-p21.2 includes the HLA region, resulting in homozygous genotypes in RCB0027 and RCB1635 that are different from that of RCB1897.

The number of chromosomes for the three sublines in this study was estimated from the SNP array profiles, which were compared with karyotypes presented in four previous studies^8–10,19^ (Table S5). Chromosomes 1, 8, 17, and 19 have three copies, while chromosomes 3, 13, and 14 have two copies. This pattern is consistent across all seven sublines. This implies that these seven chromosomes may stabilize in the K-562 genomes and could serve as consistent cytogenetic markers for K-562. It is noted that chromosomes 3, 13, and 14 exhibit LOH, indicating that one of the chromosomes was lost after triploidization. The number of specific chromosomes is unique to each lineage, demonstrating the heterogeneity of K-562 at the chromosome level.

### Sequence variants

The sequencing coverage and quality statistics for each sample are summarized in Supplementary Table S6. The CCP panel detected 68 non-synonymous variants excluding common polymorphic changes, which are listed in Table S7-1 along with their corresponding variant frequencies. When the number of filtered coverages is reduced to 20, the variants that are filtered in remain the same as with a coverage of 40. As a hotspot mutation, the *TP53* mutation is homozygous in all three cell lines. To estimate the variant allele status, the focus is on variants that differ in frequency between sublines, which are then compared with the DNA copy numbers (Table S8). The *NFE2L2* gene is subject to LOH in RCB1897, as evidenced by its variant frequency of 100%. Frequencies ranging around 33% and 66% with three copies indicate the presence of one or two copies of variants, except in the case of *NSD2* in RCB1897, which would show a mosaic pattern. Frequencies around 50% indicate the presence of one or two variants in either two or four copies. Out of the 68 variants, RCB0027, RCB1635 and RCB1897 have 17, 20 and 12 specific variants, respectively. Although 14 variants were detected in the three K-562 sublines, which could be considered a common signature of the K-562 cell line, the variant profiles are unique to each subline.

Taking into account allelic ratios^24^, the frequency of variants can be classified into five groups (Table S9). Based on this classification, the distribution of variant frequencies in each K-562 cell line is presented in Fig. S5. The combined ratios of 1:2 and 2:1 are 55%, 59.7%, and 55.4% in RCB0027, RCB1635, and RCB1897, respectively. This demonstrates that these three K-562 sublines have a similar variant signature, suggesting that the genome of RCB1897 is pseudo-triploid.

Analysis of the HBB gene revealed a synonymous mutation that was detected heterozygously in RCB0027 and RCB1635, and homozygously in RCB1897 (Table S7-2). This difference is reflected by the LOH on the short arm of chromosome 11, which occurred only in RCB1897 (Table S4). It is reported that the same variant is detected in 20% of healthy individuals^25^, indicating that it is not pathogenic but rather one of the SNPs.

### Transcriptome profiling

The *BCR-ABL1* fusion gene was confirmed using the Oncomine myeloid panel, and its expression level was comparable across all three K-562 sublines (Table S10). Furthermore, *NUP214-XKR3* was detected in all three sublines, albeit with varying expression levels.

**Figure 2:**
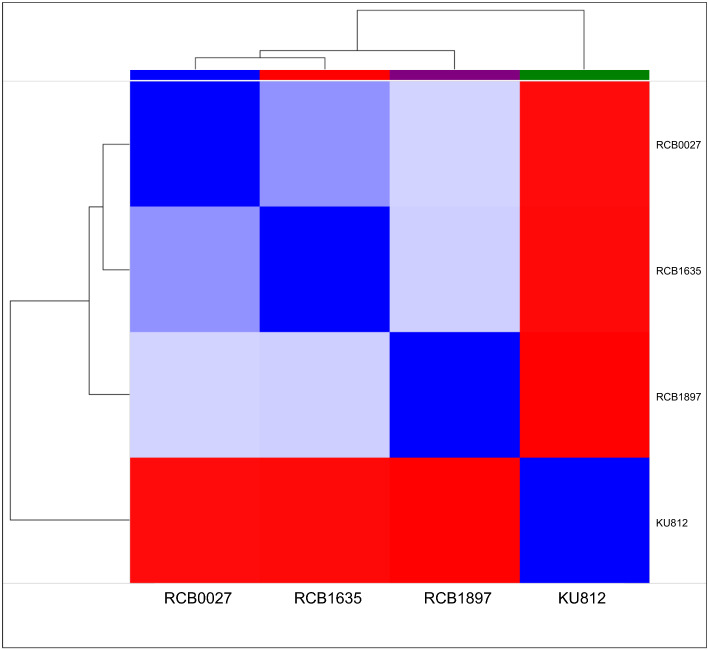
Distance matrix of three K-562 sublines and KU812. Based on transcriptome profiling, the maximal distance is denoted by a deep red color. The expression pattern indicates that RCB0027 and RCB1635 are in close proximity.

Global expression patterns were compared among the three K-562 cell lines and KU812, which was used as an outgroup for comparison. The distance matrix illustrates close similarities between RCB0027 and RCB1635, whereas RCB1897 is slightly distinct from the other two lines (Fig. 2). Principal Component Analysis (PCA) showed that the three K-562 cell lines could be distinguished from each other based on their similar PCA1 levels, and they were distinct from KU812 (Fig. S6). This suggests that the variations and commonalities among the three K-562 samples can be further explored in these datasets.

The top 100 genes that are commonly upregulated in the three K-562 cell lines are displayed through hierarchical clustering (Fig. S7A) and listed in Table S11A. *HBE1* and *HBZ* are not expressed in KU812, but they are expressed in the three K-562 cell lines. In contrast, *HBB* is one of the 30 genes that are not expressed in the three K-562 cell lines but are expressed in KU812 (Fig. S7B, Table S11B). A comparison was made between the three identified upregulated genes (13, 10, and 28) and downregulated genes (9, 8, and 45) in RCB0027, RCB1635, and RCB1897, respectively (Table 2, Fig. S7C–H, and Table S11C–H). The downregulated genes corresponded to genes that are commonly upregulated in other K-562 cells. The greater number of differentially expressed genes (DEGs) in RCB1897 is evident in the distance matrix that reflects the global expression patterns of the three analyzed sublines. Expression patterns related to gene amplification show that *ABL1* and *NUP214* exhibit a high level of expression in the 9q34 region (Fig. S7I, Table S11I). Within the 22q11.2 region, PRAME exhibits high expression levels in the three K-562 cell lines, but is not expressed in KU812 (Fig. S7J, Table S11J). Except for *HLA-E*, which was highly expressed in RCB1635, none of the HLA genes were expressed in the three K-562 cell lines (Fig. S7K, Table S11K).

**Table 2:** Number of differentially expressed genes among three K-562 sublines.

	Up-regulated	Down-regulated
RCB0027	13	9
RCB1635	10	8
RCB1897	28	45

### Hemoglobin syntheses

Cell pellets of RCB0027 and RCB1635 treated with NaB turned red, while those of RCB1897 remained white (Fig. S8). This indicates that hemoglobin was synthesized in RCB0027 and RCB1635 cells, but not in RCB1897 cells.

## Discussion

**Figure 3:**
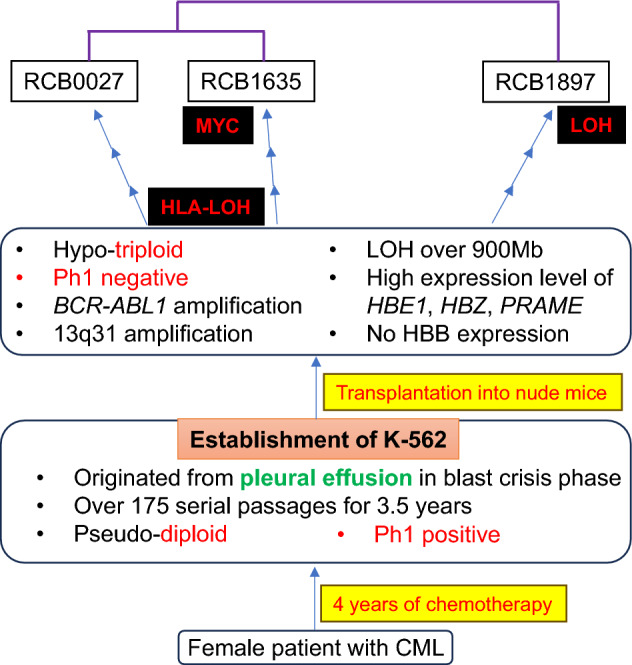
K-562 Fact Sheet, illustrating its fundamental cell data. The data consist of three parts: the clinical data, the establishment of the cell line, and current features. Differences between cellular characteristics at the establishment and the present are indicated in red. RCB0027 and RCB1635 exhibit loss of heterozygosity (LOH) in the HLA region. Additionally, RCB1635 harbors a cryptic gain in the MYC region, while RCB1897 is marked by extensive LOH. These alterations are identified based on shared characteristics. Relationships among the three sublines are shown in purple lines based on hierarchical clustering of these transcriptomic profiles. Three K-562 cell lines require unique ID numbers to distinguish them from each other and from other K-562 sublines.

A comparison of the three K-562 cell lines has demonstrated that these K-562 genomes have diverged from their original state and now exhibit distinct features. This is summarized in Table 3 and shown in Fig. 3. K-562 genomes may vary among laboratories, even if the cells have been confirmed as K-562 through conventional STR analysis. The currently available K-562 cell lines could be considered derivative sublines, despite the same cell name. This indicates that attention is required when using the original cell names for cell lines that have been exposed to extended culture after establishment, as cellular characteristics may change during cell culture.

**Table 3:** Comparison of three K-562 sublines.

	RCB0027		RCB1635	RCB1897
Ploidy	Pseudo-triploid			
Philadelphia chromosome	Lost			
*BCR-ABL1* fusion	Amplification			
X chromosome	2		2/3	1
	LOH			
Total LOH size (Mb)	977	913		1194
HLA	LOH			Hetero
Hemoglobin synthesizes	+			−
Hemoglobin genes	*HBE1*, *HBZ*; highly expressed

The K-562 genome is characterized by the presence of the *BCR-ABL1* fusion gene, which serves as a crucial molecular marker in the diagnosis and treatment of CML in clinical practice^26^. The Philadelphia chromosome was lost in K-562 due to rearrangements during cell culture^8–10,19^. On the other hand, the amplification of the *BCR-ABL1* fusion gene was reported^20,21^. The profiles of *BCR-ABL1* amplification are identical among the three sublines, despite the different genetic drift observed in each lineage. The amplification of the *BCR-ABL1* gene corresponded to an upregulation of *BCR-ABL1* mRNA expression in all three sublines. This confirms that the *BCR-ABL1* protein is accelerating the proliferation of K-562 cells.

Genomic alterations occur through tumor evolution in vitro as well as in vivo^27^. K-562 genome has undergone significant alterations since its establishment through cell culture. Differences between the K-562 genomes have arisen during cell culture, giving rise to independent K-562 lineages. RCB1635, one of the K-562 sublines deposited from the University of Tennessee Medical Center where Lozzio established K-562^1^, also showed hypo-triploid. This was different from the diploid that Lozzio reported in 1975, assuming that the ancestral K-562 clone is now extinct. It is noted that the characteristics of the ancestral K-562 are conserved separately in the three sublines (Table 3). This indicates that genomic changes occurred independently in each cell lineage and that the original properties cannot be explained by a single subline.

Zhou et al. performed extensive analysis of the K-562 genome using a variant of the K562 cell line exclusively prepared for the ENCODE project^11^. Some sequence variants detected in the ENCODE cell line were found to overlap with our results; however, variant frequencies of *ROS1* demonstrated various differences between the ENCODE project and the three sublines in our study. Furthermore, the *NOTCH1* variant was detected in two of the three sublines we studied. Although multiple deletions and duplications were identified within the *FHIT* gene^11^, a cryptic deletion in the *FHIT* region was only detected in RCB1897 (Fig. S4B). In addition, the ENCODE K-562 study showed a highly complex copy number profile for chromosome 18, which differed from any of the three sublines (Fig. S4A). This implies that K-562 can no longer be described based on the analysis of a single sample, and a comparison of multiple sublines is required to accurately characterize cell lines.

Karagiannis et al. (2023) reported an extensive characterization of their K-562 cell line following a prolonged culture^19^. New abnormal chromosomes resulting from long-term culture have been identified in the K562 karyotype^19^; however, analysis of additional changes would not serve as a reliable reference data for the K-562 cell line. Incorrect conclusions are also drawn when they state that their K562 cells retained the Philadelphia chromosome^19^ due to a misinterpretation of data. In addition, their expression analysis was performed using publicly available data, which was obtained from the analysis of the ENCODE K-562 cell line. Our study demonstrates that K-562 cells are not necessarily identical even when they share the same name. This is exemplified by comparing our K-562 sublines with the one used by the ENCODE project. Attention is required not only for cellular materials but also for data from cell lines under the same sample name.

K-562 was established from the pleural fluid of a patient in CML blast crisis after four years of chemotherapy^1^. Although K-562 is one of the 100 cell lines selected for hematological cancer research on the LL-100 panel^28^, it is important to note that K-562 differs from typical clinical CML samples. Given that K-562 is an atypical cell line for CML, it is advisable to use alternative cell lines for an in vitro CML model. However, cellular models for leukemia are limited partly due to the decreasing establishment of new cell lines, as indicated by a review of leukemia-lymphoma cell lines^29^. Further studies on leukemia require more cellular models accompanied by accurate characterization.

Taking advantage of an immortalized cell line that grows well and can be used repeatedly, K-562 can be employed as a cellular model for studying erythroid differentiation, which involves hemoglobin synthesis. K-562 cells originated from an adult patient; however, they do not synthesize adult hemoglobin. Instead, they only produce embryonic types^30,31^. Hemoglobin-related genes, *HBA2, HBG1, HBG2, HBE1*, and *HBZ*, were highly expressed in the three K-562 sublines; however, no *HBB* expression was observed. This is consistent with the previous study, which reported the expression of alpha-, gamma-, epsilon-, and zeta-globins in K562 cells, while no beta-globin production was observed^32^. In contrast, another human erythroid progenitor cell line, HUDEP-2 (RCB4557)^33^, expresses adult-type hemoglobin and is therefore widely used for various studies.

K-562 has another unique feature in that it serves as a target for NK cells^34^. It is unlikely that the K-562 cells had these characteristics before being established as a cell line. Instead, this property would result from changes that occurred during cell culture. The efficiency of erythroid differentiation and sensitivity to NK cells could depend on the in vitro environment, which is influenced by culture conditions. The three K-562 sublines in this study are each maintained using different media (Table 1), which may account for differences in cellular phenotypes. The impact of culture medium on K-562 cells will be explored in a future study, potentially revealing the molecular intricacies of genotype–phenotype correlations.

It is inevitable that some cells are altered as they proliferate. If those cells have a growth advantage, they become dominant, resulting in the replacement of the cell population, which can be explained by in vitro clonal evolution^27^. Accumulation of alterations through these processes could lead to unintentional changes in characteristics during cell culture. Based on this fact, it is advisable to use cell lines within a shorter period of culture, avoiding excessive passages. While long-term culture may occasionally serve to develop a subline with a new feature tailored for a specific purpose. Practical advice is provided on preserving frozen cell stocks immediately after resuscitation of cells. This allows for a comparison of cells before and after long-term culture to assess changes in cell lines during culture. When cellular characteristics have changed during culture, the cell name needs to be modified to distinguish it as a derivative subline. For the three K-562 sublines presented here, they need to be described along with their unique cell ID numbers: K-562_RCB0027, K562_RCB1635, and K-562_RCB1897. This is necessary to distinguish them from the original K-562 cell line and from other K-562 sublines.

In order to establish reliable reference data for commonly used cell lines such as K-562, including their sublines, it is strongly recommended that cells should be obtained from proper cell registries but never from neighboring laboratories. As sublines can be characterized by common components and unique variations, a comparison of multiple sublines is required when available. Reference profiles presented here for K-562 cells can be used to determine whether results obtained from other K-562 cells are derived from their original characteristics or from any additional alterations.

## Materials and methods

### Cell lines and DNA/RNA preparations

Three K-562 cell lines, RCB0027, RCB1635, and RCB1897, registered with the RIKEN cell bank, were employed for comparison (Table 1). These three cell lines were deposited through different routes, resulting in each having a unique culture history since their establishment. RCB0027 was deposited by the user in Japan in 1986, while RCB1635 was directly deposited in 2000 by the institution that established K-562. RCB1897 was transferred in 2004 from the Cell Resource Center for Biomedical Research for Tohoku University, which had received it from a domestic user. Another *BCR-ABL1* positive CML cell line, KU812 (RCB0495), was used for comparison in transcriptome analysis. Genomic DNA and total RNA were extracted using the AllPrep DNA/RNA Mini Kit (Qiagen, 80204). First strand cDNA was generated using the PrimeScript™ 1st strand cDNA Synthesis Kit (Takara, 6110A). All experiments were performed with mycoplasma-free cells.

### STR profiling analysis

STR markers were amplified using the GenePrint® 24 System (Promega, B1870) and analyzed by the 3500 Genetic Analyzer and GeneMapper Software 5 (Applied Biosystems).

### Chromosome analysis

Metaphase chromosomes were prepared following the standard protocol. The number of chromosomes was counted from 50 metaphases stained with Giemsa.

### Microarray analysis

DNA copy number and genotyping were examined using a CytoScan HD Array, according to the manufacturer’s protocol (Thermo Fisher Scientific, 901835). The data analyses were carried out with the hg19 reference using the Chromosome Analysis Suite software, ChAS 4.2 (Thermo Fisher Scientific).

### Sequence analysis

Target sequencing for major tumor-related genes was conducted using the Ion AmpliSeq™ Comprehensive Cancer Panel (Thermo Fisher Scientific, 4477685), which covers all exons of 409 genes. The HBB gene was analyzed using a custom panel that included 5 amplicons designed by the AmpliSeq Designer. Gene expressions were examined using the Oncomine™ Myeloid Research Assay (Thermo Fisher Scientific, A36941) and the Ion AmpliSeq™ Transcriptome Human Gene Expression Panel (Thermo Fisher Scientific, A31446). Sequence libraries and templates were prepared using the Ion AmpliSeq Kit for Chef DL8 (Thermo Fisher Scientific, A29024), followed by the Ion 510™ & Ion 520™ & Ion 530™ Kit-Chef or Ion 540™ Kit-Chef (Thermo Fisher Scientific, A34461/A30011). Sequencing was performed on the Ion GeneStudio S5 System using the Ion 530 or 540 chip (Thermo Fisher Scientific, A27763/A27765). Reads were aligned to the hg19 reference or the hg19 AmpliSeq Transcriptome ERCC v1 reference. The sequence variants and fusion genes were analyzed using the Ion Reporter 5.20 (Thermo Fisher Scientific). The expression analysis was performed using the Transcriptome Analysis Console 4.0.2 (Thermo Fisher Scientific). Variant analysis of the sequence data was performed with the settings as shown in Table S1.

### Erythroid differentiation

Each K-562 cell line (RCB0027, RCB1635, and RCB1897) was seeded in 100 mm culture dishes at a density of 1 × 10^6^ cells/mL. After treating the cells with sodium butyrate (NaB, Sigma, B5887) at a final concentration of 1 mM for 96 h, they were collected in a conical tube by centrifugation.

### Supplementary Information

Supplementary Information 1.

Supplementary Table 11.

## Data Availability

The microarray data generated in this study have been submitted to the NCBI BioProject database under accession number PRJNA737186. All other relevant data can be found within the article and its supplementary information.
