# Supplementary material for: Variable characteristics overlooked in human K-562 leukemia cell lines with a common signature
Source: Sci Rep. 2024 Apr 26;14:9619. doi: 10.1038/s41598-024-60271-8 (PMC11053119; doi:10.1038/s41598-024-60271-8)
Supplement: Supplementary file 1 — Supplementary Information 1. [file 41598_2024_60271_MOESM1_ESM.pdf]

Supplementary materials

Variable characteristics overlooked in human K-562 leukemia cell lines with a common signature.

Fumio Kasai, Kumiko Mizukoshi, Yukio Nakamura

Table S1. Filter settings for variant analysis.
Table S2. Comparison of STR profiles for 24 loci.
Table S3. Copy number alterations detected by microarray.
Table S4. LOHs detected by SNP microarray.
Table S5. Comparison of chromosome copy number between previous studies.
Table S6. Coverage data from AmpliSeq CCP panel.
Table S7. Sequence variants and their frequencies.
Table S8. Allelic status of sequence variants.
Table S9. Number of variants sorted by allelic frequency.
Table S10. Fusion genes detected by Oncomine Myeloid panel.
Table S11. List of genes filtered in transcriptome analysis. (Excel file)

Figure S1. Electropherograms of 24 STR markers.
Figure S2. Examples of a G-banding karyotypes.
Figure S3. Whole genome profiles from SNP microarray analysis.
Figure S4. Cryptic DNA copy number changes detected by SNP microarrays.
Figure S5. Distribution of variant frequencies.
Figure S6. Principal component analysis of global gene expression patterns.
Figure S7. Heatmaps representing the relative expression levels over the four samples.
Figure S8. Hemoglobin synthesis induced by sodium butyrate.

Table S1. Filter setting for variant analysis.

Allele Frequency	>= 0.2
Filtered Coverage	>= 40
PValue	<= 0.01
Minor Allele Frequency	<= 0.001
UCSC Common SNPs	Not In
Location in	exonic
Variant Effect in	missense,<br>nonframeshiftInsertion,<br>nonframeshiftDeletion,<br>nonframeshiftBlockSubstitution,<br>nonsense,<br>stoploss,<br>frameshiftInsertion,<br>frameshiftDeletion,<br>frameshiftBlockSubstitution

Table S2. Comparison of STR profiles for 24 loci.

Data for JCRB and DSMZ are obtained from the JCRB cell line authentication database. Locus in bold indicates differences between sublines, suggesting changes in repeat numbers during cell culture.

STR marker		Putative origin		K-562 subline													
Locus	Position			RIKEN								ATCC			DSMZ		
				RCB0027		RCB1635			RCB1897			CCL-243			ACC-10		
**D1S1656**	1q42	15	16	15	16	**14**	15	16	15	16							
TPOX	2p25.3	8	9	8	9	8	9		8	9		8	9		8	9	
**D2S441**	2p14	10	14	10	14	10	14		10								
D2S1338	2q35	17		17		17			17			17					
D3S1358	3p21.3	16		16		16			16								
**FGA**	4q28	21	24	21	24	21	24	**25**	21	24		21	24				
**D5S818**	5q23.2	11	12	11	12	11	12	**13**	11	12		11	12		11	12	
CSF1PO	5q32	9	10	9	10	9	10		9	10		9	10		9	10	
D7S820	7q21.11	9	11	9	11	9	11		9	11		9	11		9	11	
D8S1179	8q24.1	12		12		12			12			12					
D10S1248	10q26.3	12		12		12			12								
TH01	11p15.5	9.3		9.3		9.3			9.3			9.3			9.3		
vWA	12p13.31	16		16		16			16			16			16		
**D12S391**	12p13.2	23		**22**	23	23			23								
D13S317	13q31.1	8		8		8			8			8			8		
**Penta_E**	15q26.2	5	14	5	14	5			5	14		5	14				
**D16S539**	16q24.1	11	12	11	12	11	12	**13**	12			11	12		11	12	**13**
**D18S51**	18q21.3	15	16	15	16	15	16		16			15	16				
**D19S433**	19q12	14	14.2	14	14.2	14	14.2		14.2			14	14.2				
**D21S11**	21q21.1	29	31	**30**	31	29	**30**	31	29	**30**	31	29	**30**	31			
Penta_D	21q22.3	9	13	9	13	9	13		9	13		9	13				
D22S1045	22q12.3	16		16		16			16								
Amelogenin	Xp22.2/Yp11.2	X		X		X			X			X			X		
DYS391	Yq11.21																

Table S3. Copy number alterations detected by microarray.

Chromosome	RCB0027	RCB1635	RCB1897
1	arr[GRCh37] 1p36.33p13.3(849467-107314000)x3<br>arr[GRCh37] 1p13.3p12(107314078-120524145)x4<br>arr[GRCh37] 1q21.1q23.3(144376265-162374710)x3<br>arr[GRCh37] 1q23.3q32.2(162374711-210300490)x4<br>arr[GRCh37] 1q32.2(210305936-210831445)x3<br>arr[GRCh37] 1q32.2q44(210831550-248636951)x4	arr[GRCh37] 1p36.33p32.3(849467-55308573)x3<br>arr[GRCh37] 1p21.1p13.3(105433702-105561371)x3-4<br>arr[GRCh37] 1p13.3p12(107314078-120524145)x5<br>arr[GRCh37] 1q21.1 q32.2(144376265-210300490)x3<br>arr[GRCh37] 1q32.2q41(210831550-215167579)x3<br>arr[GRCh37] 1q41q42.13(215175063-229608165)x4-5<br>arr[GRCh37] 1q42.13q44(229611914-248636951)x3	arr[GRCh37] 1p36.33p13.3(849467-107314000)x3<br>arr[GRCh37] 1p13.3p12(107314078-120524145)x4<br>arr[GRCh37] 1q21.1q23.3(144376265-162374710)x3<br>arr[GRCh37] 1q23.3q44(162374711-248636951)x4
2	arr[GRCh37] 2p25.3q24.3(12770-168842046)x3<br>arr[GRCh37] 2q24.3q32.1(168842102-188684740)x4<br>arr[GRCh37] 2q35(215311414-217833891)x3<br>arr[GRCh37] 2q37.1(233301638-235079622)x3	arr[GRCh37] 2p25.3q24.1(12770-155575272)x3<br>arr[GRCh37] 2q24.1q32.1(155575571-188684740)x4<br>arr[GRCh37] 2q33.1q37.2(201937392-236090444)x3<br>arr[GRCh37] 2q37.2(236091095-236910990)x5<br>arr[GRCh37] 2q37.2q37.3(236911246-242703758)x3	arr[GRCh37] 2p25.3p16.3(12770-51754782)x3<br>arr[GRCh37] 2p12q37.3(82654218-242703758)x3
3	arr[GRCh37] 3p26.3p21.31(61892-48218497)x4	arr[GRCh37] 3p26.3p21.31(61892-48218497)x4	arr[GRCh37] 3p26.3p21.31(61892-48218497)x2-3<br>arr[GRCh37] 3p14.2(60488133-60544450)x0<br>arr[GRCh37] 3p13p11.1(71129074-90502849)x1
4	arr[GRCh37] 4p16.3q32.1(68346-160488595)x3<br>arr[GRCh37] 4q32.1q32.2(160493173-163616036)x1<br>arr[GRCh37] 4q32.2q35.1(163624025-186308406)x3<br>arr[GRCh37] 4q35.1q35.2(186308446-190957473)x4	arr[GRCh37] 4p16.3q32.1(68346-160488595)x3<br>arr[GRCh37] 4q32.1q32.2(160493173-163616036)x1<br>arr[GRCh37] 4q32.2q35.2(163624025-190957473)x3	arr[GRCh37] 4p15.33q13.1(14771119-64308401)x3<br>arr[GRCh37] 4q21.22q32.1(83325038-160488595)x3<br>arr[GRCh37] 4q32.1q32.2(160493173-163616036)x1<br>arr[GRCh37] 4q32.2q35.2(163624025-190957473)x3
5	arr[GRCh37] 5p15.33p11(113577-46273389)x4<br>arr[GRCh37] 5q11.1q35.3(50669229-180719788)x3	arr[GRCh37] 5p15.33q35.3(113577-180719788)x3	arr[GRCh37] 5p15.33p11(113577-46273389)x4<br>arr[GRCh37] 5q11.1q35.3(50669229-180719788)x3
6	arr[GRCh37] 6p25.3p22.3(156975-16771601)x4<br>arr[GRCh37] 6p22.3p21.32(16771685-32649020)x3<br>arr[GRCh37] 6p21.32p21.1(32649307-43276943)x4<br>arr[GRCh37] 6p21.1p12.3(43276944-51758790)x4-5<br>arr[GRCh37] 6p12.3q22.1(51766080-116476608)x3	arr[GRCh37] 6p25.3p21.2(156975-38321528)x3<br>arr[GRCh37] 6p21.2p12.3(38321556-51758790)x4<br>arr[GRCh37] 6p12.3q23.3(51766080-135767685)x3<br>arr[GRCh37] 6q23.3q27(136767925-170117372)x4	arr[GRCh37] 6p25.3p21.2(156975-38321528)x3<br>arr[GRCh37] 6q23.3q27(136767925-170117372)x3

	arr[GRCh37] 6q22.1q22.31(116476659-118824847)x4<br>arr[GRCh37] 6q22.31(118826254-121544998)x3<br>arr[GRCh37] 6q22.31q27(161407819-170117372)x3		
7	arr[GRCh37] 7p14.1(41065646-43037284)x4<br>arr[GRCh37] 7p14.1q22.3(43044439-104605103)x3<br>arr[GRCh37] 7q22.3q36.3(104605365-159119707)x4	arr[GRCh37] 7q11.21q22.3(62437463-104605103)x3<br>arr[GRCh37] 7q22.3q36.3(104605365-159119707)x4	arr[GRCh37] 7q11.21q11.23(62437463-76835389)x3<br>arr[GRCh37] 7q11.23q36.3(76835813-159119707)x3-4
8	arr[GRCh37] 8p23.3q24.3(585868-146295770)x3	arr[GRCh37] 8p23.3p23.1(585868-11743764)x4<br>arr[GRCh37] 8p23.1q24.3(11744741-146295770)x3	arr[GRCh37] 8p23.3p11.23(585868-37783155)x4<br>arr[GRCh37] 8p11.23q24.3(37783175-146295770)x3
9	arr[GRCh37] 9p21.3p21.2(20314007_26590984)x0<br>arr[GRCh37] 9p21.1(28560173-31608841)x0<br>arr[GRCh37] 9p21.1p13.2(31609353-37064086)x3<br>arr[GRCh37] 9p13.2p13.1(37064496-38434954)x5<br>arr[GRCh37] 9q22.31q34.12(94049755-133605551)x3<br>arr[GRCh37] 9q34.12q34.13(133605552-134141691)x12-15<br>arr[GRCh37] 9q34.13-q34.3(134273127-141020388)x4	arr[GRCh37] 9p21.3p21.2(20314007_26590984)x0<br>arr[GRCh37] 9p21.2p21.1(26585558-28563340)x1<br>arr[GRCh37] 9p21.1(28560173-31608841)x0<br>arr[GRCh37] 9p13.2p13.1(37064496-38434954)x3<br>arr[GRCh37] 9q22.31q34.12(94049755-133605551)x3<br>arr[GRCh37] 9q34.12q34.13(133605552-134141691)x12-15<br>arr[GRCh37] 9q34.13-q34.3(134273127-141020388)x3	arr[GRCh37] 9p24.3p21.3(203861-20756546)x1<br>arr[GRCh37] 9p21.3p21.2(20314007_26590984)x0<br>arr[GRCh37] 9p21.1(31609353-31973082)x1<br>arr[GRCh37] 9p13.3p13.2(35704970-37070371)x1<br>arr[GRCh37] 9p13.2p13.1(37064496-38434954)x3<br>arr[GRCh37] 9q22.31q22.33(94049755-102213748)x2-4<br>arr[GRCh37] 9q22.33q31.3(102214399-113398560)x4<br>arr[GRCh37] 9q31.3q34.12(113399297-133605551)x3<br>arr[GRCh37] 9q34.12q34.13(133605552-134141691)x12-15<br>arr[GRCh37] 9q34.13-q34.3(134273127-141020388)x3
10	arr[GRCh37] 10p15.1p11.1(100047-39070794)x3<br>arr[GRCh37] 10q11.21(42443239-87846719)x4	arr[GRCh37] 10p15.1p11.1(100047-39070794)x3<br>arr[GRCh37] 10q11.21(42443239-87846719)x4	arr[GRCh37] 10p15.1q23.2(100047-87846719)x3
11	arr[GRCh37] 11p15.5q25(230616-134938469)x3	arr[GRCh37] 11p15.5q25(230616-134938469)x3	arr[GRCh37] 11q11q25(54701511-134938469)x3
12	arr[GRCh37] 12p12.1q24.33(22707436-133777901)x3	arr[GRCh37] 12p13.33q24.31(173786-125636786)x3<br>arr[GRCh37] 12q24.31q24.32(125636787-128094452)x4<br>arr[GRCh37] 12q24.32q24.33(128016481-133777901)x3	arr[GRCh37] 12p12.1(22707436-25391470)x4<br>arr[GRCh37] 12p12.1q24.33(25391510-133777901)x3
13	arr[GRCh37] 13q31.1(81089543-81468761)x7<br>arr[GRCh37] 13q31.3(90436445-92475630)x8<br>arr[GRCh37] 13q31.3(92953101-93348248)x8-9<br>arr[GRCh37] 13q31.3(93852315-94025367)x8-9	arr[GRCh37] 13q31.1(81089543-81468761)x6<br>arr[GRCh37] 13q31.3(90436445-92475630)x6<br>arr[GRCh37] 13q31.3(92953101-93348248)x6<br>arr[GRCh37] 13q31.3(93852315-94025367)x6	arr[GRCh37] 13q31.1(81089543-81468761)x8<br>arr[GRCh37] 13q31.3(90436445-92475630)x8<br>arr[GRCh37] 13q31.3(92953101-93348248)x8<br>arr[GRCh37] 13q31.3(93852315-94025367)x8

	arr[GRCh37] 13q33.3(108492593-108660007)x7	arr[GRCh37] 13q33.3(108492593-108660007)x5	arr[GRCh37] 13q33.3(108492593-108660007)x7
14	arr[GRCh37] 14q12(30631930-33191224)x3		
15	arr[GRCh37] 15q11.2q15.1(22770588-42467665)x3	arr[GRCh37] 15q11.2q26.1(22770588-91981115)x3	arr[GRCh37] 15q11.2q26.3(22770588-102429111)x3
16	arr[GRCh37] 16p13.3(85880-563921)x3<br>arr[GRCh37] 16p13.3(565160-1390929)x3-4<br>arr[GRCh37] 16p13.3q23.1(1391154-78257274)x3<br>arr[GRCh37] 16q23.1q24.1(78385090-85644397)x4<br>arr[GRCh37] 16q24.1q24.2(85644396-88219734)x3<br>arr[GRCh37] 16q24.2q24.3(88222733-90155062)x4	arr[GRCh37] 16p13.3(85880-563921)x3<br>arr[GRCh37] 16p13.3(565160-1390929)x3-4<br>arr[GRCh37] 16p13.3q23.1(1391154-78257274)x3<br>arr[GRCh37] 16q23.1q24.1(78385090-85644397)x4<br>arr[GRCh37] 16q24.1q24.2(85644396-88219734)x3<br>arr[GRCh37] 16q24.2q24.3(88222733-89222690)x4<br>arr[GRCh37] 16q24.3(89223224-90155062)x3	arr[GRCh37] 16p13.3(565160-1390929)x2-3<br>arr[GRCh37] 16q11.2q23.1(1391154-78257274)x3<br>arr[GRCh37] 16q23.1q23.2(78385090-79812812)x3-4<br>arr[GRCh37] 16q23.2q24.1(79812838-85126969)x3
17	arr[GRCh37] 17q11.2q24.2(25270397-66601449)x3<br>arr[GRCh37] 17q24.2q24.3(66601758-68375553)x3-4<br>arr[GRCh37] 17q24.3q25.3(68376788-81041937)x3	arr[GRCh37] 17q11.2 q25.3 (25270397-81041937)x3	arr[GRCh37] 17q11.2 q12 (25270397-32518944)x3<br>arr[GRCh37] 17q21.33q24.2(48937046-64653322)x3
18	arr[GRCh37] 18p11.32p11.22(136227-10435867)x5<br>arr[GRCh37] 18p11.22q12.1(10436287-28189640)x4<br>arr[GRCh37] 18q12.1q12.3(28191912-39612005)x3<br>arr[GRCh37] 18q21.32q23(56247599-78014122)x3	arr[GRCh37] 18p11.32q11.2(136227-22412926)x3<br>arr[GRCh37] 18q11.2q12.3(22414113-39569745)x4-5	arr[GRCh37] 18q11.2q12.1(24570667-25076257)x3-4
19	arr[GRCh37] 19p13.3(260912-713294)x3-4<br>arr[GRCh37] 19p13.3q13.43(713302-58956887)x3	arr[GRCh37] 19p13.3(260912-713294)x3-4<br>arr[GRCh37] 19p13.3q12(713302-31507397)x3<br>arr[GRCh37] 19q12q13.33(31507489-50272702)x4<br>arr[GRCh37] 19q13.41q13.43(51604581-58956887)x3	arr[GRCh37] 19p13.3(260912-713294)x3-4<br>arr[GRCh37] 19p13.3p11(713302-24507140)x3<br>arr[GRCh37] 19q13.41q13.43(51604581-58956887)x3
20	arr[GRCh37] 20q11.21q13.33(29420338-62915554)x3	arr[GRCh37] 20q11.21q13.33(29420338-62915554)x3	arr[GRCh37] 20p13p11.23(61569-19832869)x2-3<br>arr[GRCh37] 20q11.21q11.23(29420338-36585366)x3<br>arr[GRCh37] 20q13.33(59895770-62915554)x2-3
21	arr[GRCh37] 21q21.2(25502405-48097371)x3	arr[GRCh37] 21q21.2(25502405-48097371)x3	arr[GRCh37] 21q11.2q21.1(15006457-19609648)x4<br>arr[GRCh37] 21q21.1q21.2(19611022-25433581)x3<br>arr[GRCh37] 21q21.2q22.11(25502405-34712565)x5<br>arr[GRCh37] 21q22.11q22.3(34718662-48097371)x3

22	arr[GRCh37] 22q11.1(16888900-17285557)x7-9<br>arr[GRCh37] 22q11.1q11.21(17286146-18970561)x3<br>arr[GRCh37] 22q11.21(18970562-22040245)x7-9<br>arr[GRCh37] 22q11.21q11.23(22053831-23635638)x15-20	arr[GRCh37] 22q11.1(16888900-17285557)x6-8<br>arr[GRCh37] 22q11.1q11.21(17286146-18970561)x3<br>arr[GRCh37] 22q11.21(18970562-22040245)x8-10<br>arr[GRCh37] 22q11.21q11.23(22053831-23635638)x15-20	arr[GRCh37] 22q11.1(16888900-17285557)x6-8<br>arr[GRCh37] 22q11.1q11.21(17286146-18970561)x3<br>arr[GRCh37] 22q11.21(18970562-22040245)x8-10<br>arr[GRCh37] 22q11.21q11.23(22053831-23635638)x15-20
X	arr[GRCh37] Xq23(113171121-114371316)x3	arr[GRCh37] Xp22.33q28(169805-155233846)x2-3	arr[GRCh37] Xp22.33q26.3(169805-133605600)x1<br>arr[GRCh37] Xq26.3(133608174-133927270)x0<br>arr[GRCh37] Xq26.3q28(133937409-155233846)x1
Y	arr(Y)x0	arr(Y)x0	arr(Y)x0

Table S4. LOHs detected by SNP microarray.

Position in bold indicates LOH specific to RCB0027 or RCB1897.

<table>
<tr><th>Position</th><th>RCB0027</th><th>RCB1635</th><th>RCB1897</th></tr>
<tr><td>2p16.3-q32.1</td><td></td><td></td><td rowspan="2">182.3</td></tr>
<tr><td>2q32.1-37.3</td><td>55.3</td><td>55.3</td></tr>
<tr><td>3p26.3-q29</td><td>194.7</td><td>194.7</td><td>194.7</td></tr>
<tr><td>4q13.1-21.22</td><td></td><td></td><td>18.8</td></tr>
<tr><td>6p25.3-p21.2</td><td rowspan="2">49.5</td><td>38.0</td><td></td></tr>
<tr><td>6p21.2-p12.3</td><td></td><td></td></tr>
<tr><td>7p22.3-7p14.1</td><td>41.1</td><td></td><td></td></tr>
<tr><td>9p24.3-p21.3</td><td>20.7</td><td>20.7</td><td>20.7</td></tr>
<tr><td>9p21.1-p13.2</td><td>5.7</td><td>5.7</td><td>5.7</td></tr>
<tr><td>9q21.11-34.3</td><td>70.0</td><td>70.0</td><td>70.0</td></tr>
<tr><td>10q23.1-q26.3</td><td>47.6</td><td>47.6</td><td>47.6</td></tr>
<tr><td>11p15.5-p11.12</td><td></td><td></td><td>51.6</td></tr>
<tr><td>12p13.33-p12.1</td><td>22.7</td><td>22.7</td><td>22.7</td></tr>
<tr><td>13q11-34</td><td>115.1</td><td>115.1</td><td>115.1</td></tr>
<tr><td>14q11-32.33</td><td>107.3</td><td>107.3</td><td>107.3</td></tr>
<tr><td>16p13.3-q24.3</td><td></td><td></td><td>90.2</td></tr>
<tr><td>17p13.3-p11.1</td><td>22.2</td><td>22.2</td><td>22.2</td></tr>
<tr><td>19q11-13.43</td><td></td><td></td><td>30.8</td></tr>
<tr><td>20p13-p11.1</td><td>26.3</td><td>26.3</td><td>26.3</td></tr>
<tr><td>21q11.2-21.2</td><td>11.1</td><td></td><td></td></tr>
<tr><td>22q11.21-13.33</td><td>32.5</td><td>32.5</td><td>32.5</td></tr>
<tr><td>Xp22.33-q28</td><td>155.2</td><td>155.2</td><td>155.2</td></tr>
<tr><td>Total</td><td>977</td><td>913.3</td><td>1193.7</td></tr>
</table>

Table S5. Comparison of the number of chromosomes in three sublines and four previous studies. These counts include not only apparently normal chromosomes but also derivative chromosomes. Numbers in bold are consistent across sublines, while numbers in red indicate uniqueness within a specific subline. The X chromosome in RCB1635 is mosaic with 2 or 3 copies.

Chromosome	RCB0027	RCB1635	RCB1897	Naumann	Gribble	Karagiannis	Zhou
**1**	**3**	**3**	**3**	**3**	**3**	**3**	**3**
2	3	3	3	3	2	3	3
**3**	**2**	**2**	**2**	**2**	**2**	**2**	**2**
4	3	3	3	3	3	2	3
5	4	3	4	4	4	4	3
6	3	3	2	3	3	3	3
7	3	3	3	4	4	4	4
**8**	**3**	**3**	**3**	**3**	**3**	**3**	**3**
9	2	2	2	2	3	2	2
10	4	4	3	4	3	4	3
11	3	3	3	4	2	3	3
12	3	3	3	4	3	3	2
**13**	**2**	**2**	**2**	**2**	**2**	**2**	**2**
**14**	**2**	**2**	**2**	**2**	**2**	**2**	**2**
15	3	3	3	3	3	2	3
16	3	3	3	3	2	3	3
**17**	**3**	**3**	**3**	**3**	**3**	**3**	**3**
18	4	3	2	2	4	3	3
**19**	**3**	**3**	**3**	**3**	**3**	**3**	**3**
20	3	3	3	2	3	3	2
21	2	2	4	3	4	2	4
22	3	3	3	3	3	4	2
X	2	2 / 3	1	2	2	2	2

Table S6. Sequence statistics data.

S6-1. Coverage data from AmpliSeq CCP panel.

Sample ID	Total number of sequenced reads	Total number of uniquely mapped reads (hg19)	Total number of covered targeted bases (Mbp)	Median coverage (and range) per targeted base	Percentage of targeted bases with coverage	
					≥40	≥20
RCB0027	7,596,223	7,430,790	878.8641	504.67(0-10628)	94.78%	97.15%
RCB1635	9,012,001	8,816,957	1045.1868	600.31(0-13837)	95.07%	97.29%
RCB1897	8,833,887	8,649,707	1023.3250	587.89(0-10252)	94.96%	97.25%

S6-2. QC report from Oncomine Myeloid Panel for RNA Fusions

Sample ID	Total Mapped Fusion Panel Reads (hg19)	Fusion Sample QC	Total Unmapped Reads	Average Read Length	Expression Controls Total Reads
RCB0027	233,099	PASS, [Total Mapped Fusion Panel Reads>5000; Mean Read Length >60]	64,333	128	111,923
RCB1635	155,689	PASS, [Total Mapped Fusion Panel Reads>5000; Mean Read Length >60]	62,657	136	78,689
RCB1897	105,376	PASS, [Total Mapped Fusion Panel Reads>5000; Mean Read Length >60]	30,866	130	54,526

Table S7. Sequence variants and their frequencies.

S7-1. Analysis by Comprehensive Cancer Panel.

Genes	Coding	Amino Acid Change	Allele Frequency %		
			RCB0027	RCB1635	RCB1897
AKT3	c.109G>T	p.Gly37Ter	30.08	31.93	32.71
ASXL1	c.1773C>A	p.Tyr591Ter	28.24	29.25	26.47
CDH2	c.1034A>G	p.Tyr345Cys	34.86	28.11	39.24
CIC	c.2782G>A	p.Ala928Thr	71.11	79.77	99.73
DST	c.14427G>C	p.Met4809Ile	40.25	36.93	55.71
ERBB3	c.1831C>T	p.Arg611Trp	64.88	67.62	66.85
FANCC	c.178G>A	p.Val60Ile	100	99.67	99.92
GDNF	c.499G>A	p.Asp167Asn	54.14	32.56	51.9
IGF2R	c.835A>G	p.Ser279Gly	34.81	49.58	66.06
KAT6A	c.5629C>T	p.Arg1877Cys	32.22	26.88	38.2
NFE2L2	c.800C>G	p.Thr267Arg	48.48	44.77	100
NSD2	c.1798C>T	p.Arg600Ter	33.12	27.11	63.49
ROS1	c.1079G>T	p.Arg360Ile	47.29	34.36	49.68
TP53	c.406_407insC	p.Gln136ProfsTer13	100	99.42	100
CBL	c.2374G>C	p.Asp792His	37.02	38.34	
CRBN	c.1033C>T	p.Pro345Ser	25.39	23.55	
CRTC1	c.1274A>T	p.Gln425Leu	25.84	21.89	
NOTCH1	c.977G>T	p.Gly326Val	52.43	35.22	
HNF1A	c.1123_1124delGG, c.1124_1126delGCC	p.Gly375ProfsTer43, p.Gly375_Pro376delinsAla	CC=56.02, G=43.99		CC=59.51, G=40.49
CIC	c.3024G>C	p.Gln1008His	36.81		
CSMD3	c.10624T>A	p.Ser3542Thr	30.69		
DPYD	c.1920C>A	p.Ser640Arg	34.43		
EGFR	c.1229G>C	p.Trp410Ser	22.97		
EPHA7	c.2610G>T	p.Leu870Phe	39.18		
ERBB4	c.542delA	p.Asn181MetfsTer28	61.84		
HNF1A	c.1136delC, c.1136_1138delCTGinsTGC	p.Pro379LeufsTer5, p.[Pro379Leu;Val380Leu]	TG=95.90, TGC=4.10		
LRP1B	c.13255A>T	p.Thr4419Ser	23.96		
MSH6	c.317G>A	p.Trp106Ter	30.9		
NLRP1	c.199T>A	p.Trp67Arg	44.66		
NSD1	c.1375G>T	p.Asp459Tyr	31.41		
PKHD1	c.4593_4594insT	p.Asn1532Ter	44.09		
SGK1	c.214C>T	p.His72Tyr	35.58		
SYNE1	c.9247G>A	p.Ala3083Thr	20.6		
TAF1L	c.5234C>T	p.Ala1745Val	35.43		
TGFBR2	c.1006G>A	p.Glu336Lys	23.43		
THBS1	c.2215G>A	p.Asp739Asn	30.05		
AXL	c.1120C>T	p.Gln374Ter		25.58	
BCL11B	c.2293T>G	p.Ser765Ala		42.62	
BRIP1	c.2637_2638insG	p.Ser880ValfsTer4		59.57	
CDH2	c.1949G>T	p.Arg650Ile		24.35	
CSMD3	c.3820C>A	p.Gln1274Lys		27.56	
DDR2	c.1466G>T	p.Arg489Leu		32.96	
ESR1	c.1419C>A	p.Asp473Glu		24.17	
FANCA	c.137C>A	p.Ser46Ter		22.68	
KMT2A	c.304G>A	p.Gly102Arg		30.68	
KMT2D	c.4605G>T	p.Glu1535Asp		24.36	
LRP1B	c.1705T>A	p.Tyr569Asn		33.5	
MLLT10	c.1517C>G	p.Ser506Ter		32.47	
MMP2	c.1152C>G	p.Asp384Glu		29.57	
NIN	c.281C>A	p.Ala94Asp		57.14	
PML	c.1140G>C	p.Lys380Asn		33.62	

RALGDS	c.1506C>G	p.Asp502Glu		28.32	
SOX11	c.430G>A	p.Gly144Ser		26.13	
STK11	c.772_774delGACinsACG, c.772delG	p.Asp258Thr, p.Asp258ThrfsTer29		AC=75.41, ACG=24.59	
SYNE1	c.4430C>A	p.Ser1477Tyr		27.97	
TCF12	c.230C>T	p.Thr77Ile		31.01	
BCL9	c.2542A>G	p.Ile848Val			28.63
CBL	c.141_142delGCinsTT	p.[Pro47=;Pro48Ser]			41.32
ERBB4	c.229C>A	p.Leu77Met			67.71
JAK3	c.2089C>T	p.Arg697Trp			21.59
KDM6A	c.605T>A	p.Leu202Ter			98.04
LRP1B	c.4057G>T	p.Asp1353Tyr			34.75
MALT1	c.2439delT	p.Phe813LeufsTer20			56.39
MYH11	c.1232G>A	p.Gly411Glu			49.1
NPM1	c.134G>T	p.Arg45Ile			34.29
RNF213	c.1730_1733delCCGA	p.Thr577IlefsTer9			30.75
SYNE1	c.21247C>A	p.Gln7083Lys			28.26
TRRAP	c.89A>G	p.Asp30Gly			62.24

S7-2. HBB gene targeting sequencing.

Position	Coding	Amino Acid Change	RCB0027	RCB1635	RCB1897
chr11:5248243	c.9T>C	p.His3=	59.0	50.8	100

Table S8. Allelic status of sequence variants.

	RCB0027	RCB1635	RCB1897
Gene	Frequency (%)		
Position	Copy number		
	Allelic status		
NFE2L2	48.5	44.8	100
2q31.2	4	4	3: LOH
	○○●●	○○●●	●●●
NSD2	33.1	27.1	63.5
4p16.3	3	3	2
	○○●	○○●	○●/●●
GDNF	54.1	32.6	51.9
5p13.2	4	3	4
	○○●●	○○●	○○●●
ROS1	47.3	34.4	49.7
6q22.1	4	3	2
	○○●●	○○●	○●
IGF2R	34.8	49.6	66.1
6q25.3	3	4	3
	○○●	○○●●	○●●
NOTCH1	52.4	35.2	0
9q34.3	4	3	3
	○○●●	○○●	○○○

Table S9. Number of variants sorted by allelic frequency.

Frequency	RCB0027	RCB1635	RCB1897	Allele ratio	Estimated copy number
21.0-29.0	78	81	62	1:3	4
29.3-37.3	120	142	117	1:2	3
46.0-54.0	73	61	74	1:1	2 or 4
62.6-70.6	110	117	83	2:1	3
71.0-79.0	37	33	25	3:1	4

Table S10. Fusion genes detected by Oncomine Myeloid panel.

Genes (Exons)	Read counts per million		
	RCB0027	RCB1635	RCB1897
BCR(14) - ABL1(2)	158584.3	149493	146527.1
NUP214(29) - XKR3(4)	7006.8	12407.9	3108.1

Figure S1A. RCB0027

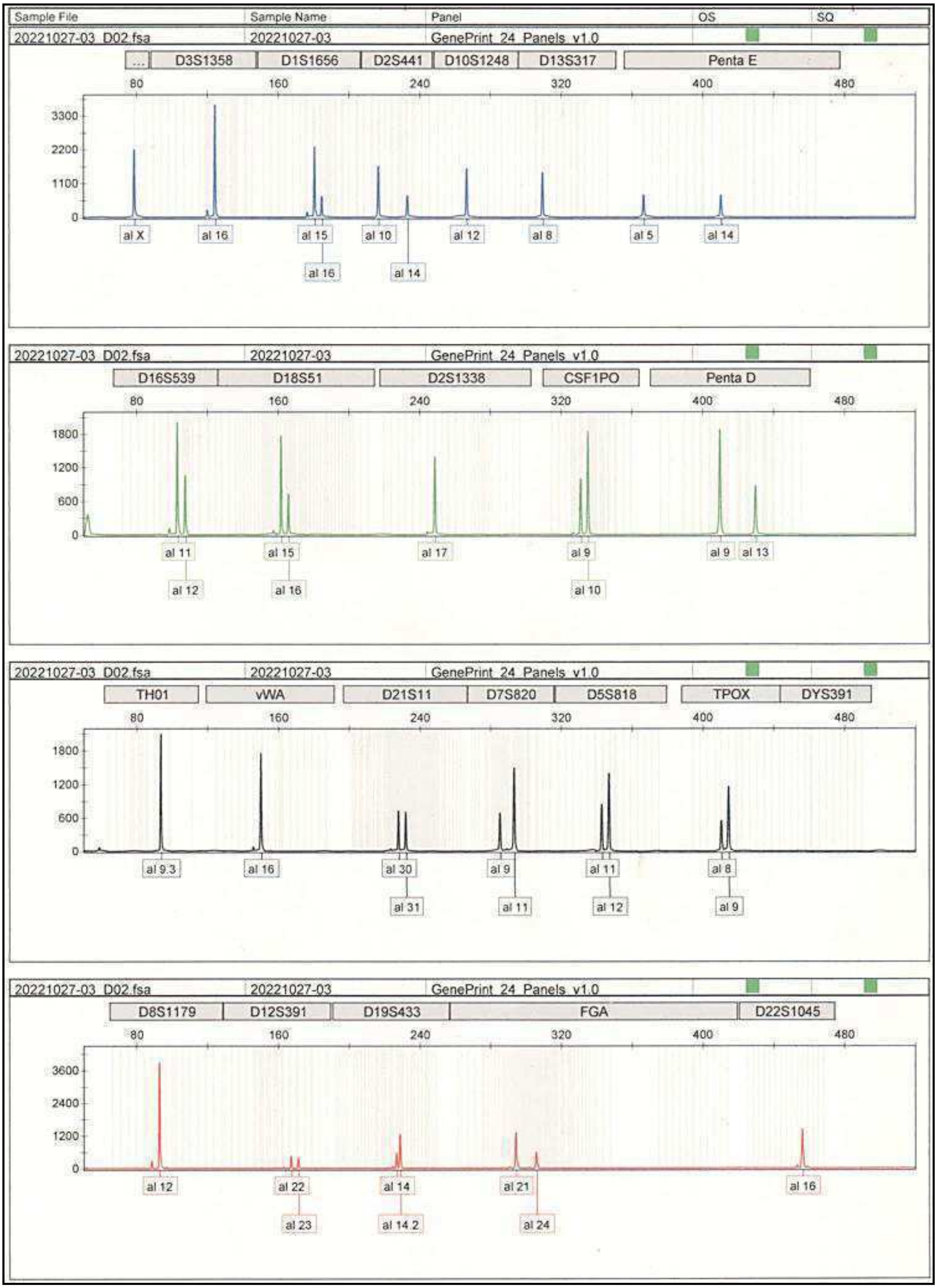

Electropherograms for 24 STR markers analyzed in RCB0027 (A), RCB1635 (B) and RCB1897 (C). As K-562 is of female origin, DYS391 is not detected, and peaks are obtained from the remaining 23 loci. The data is listed in Table S2. RCB0027 (A) exhibits two peaks at D12S391, despite the fact that the locus is encompassed within the LOH region (Figure 1). RCB1635 (B) exhibits three peaks at 5 loci, which are caused by variations in the number of repeats resulting from the insertion or deletion of a repeat unit. Fourteen out of 23 loci exhibit a single peak in RCB1897, which is reflected by extensive LOHs (Figure1, Table S3).

Figure S1B. RCB1635

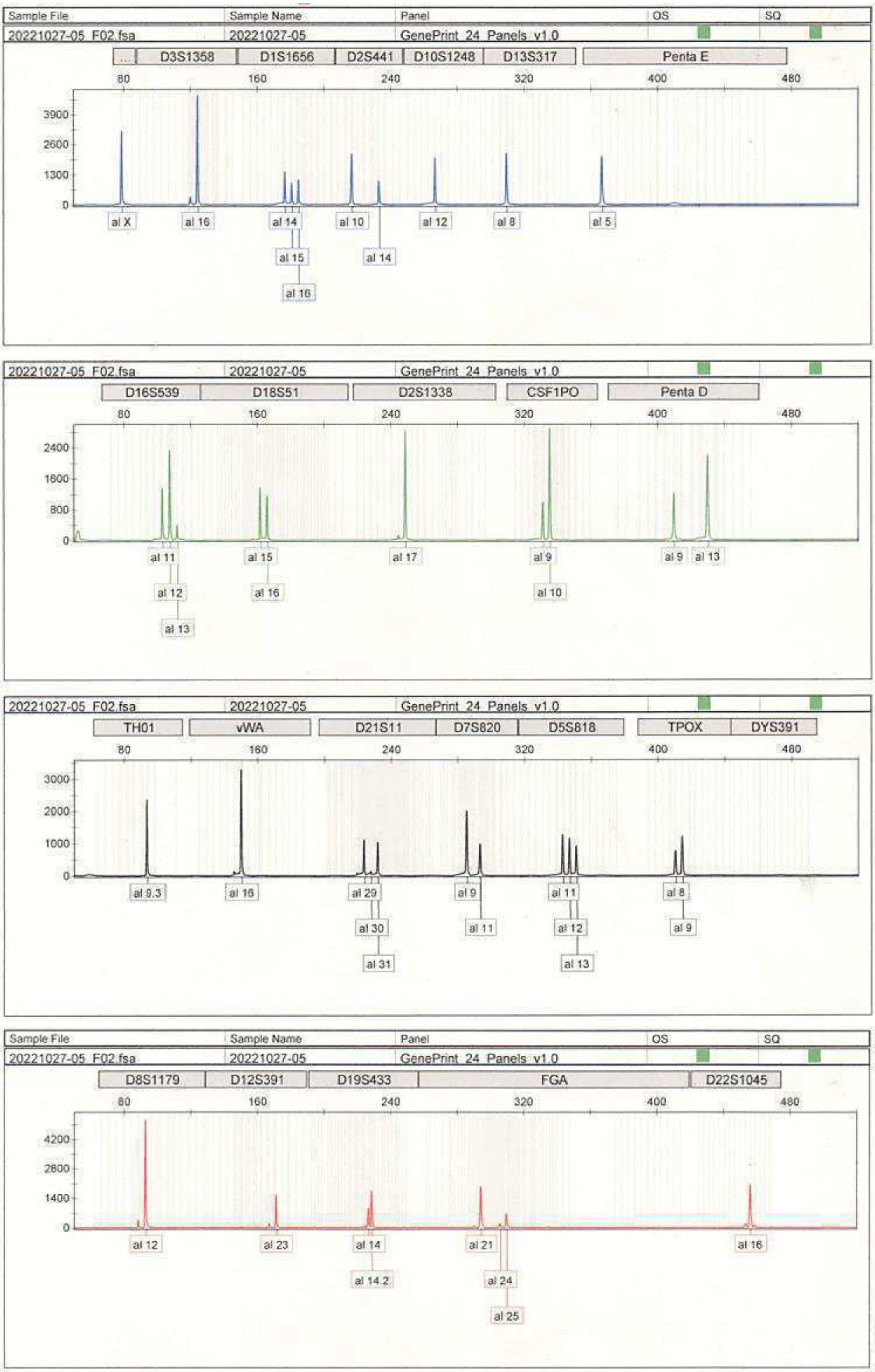

Figure S1C. RCB1897

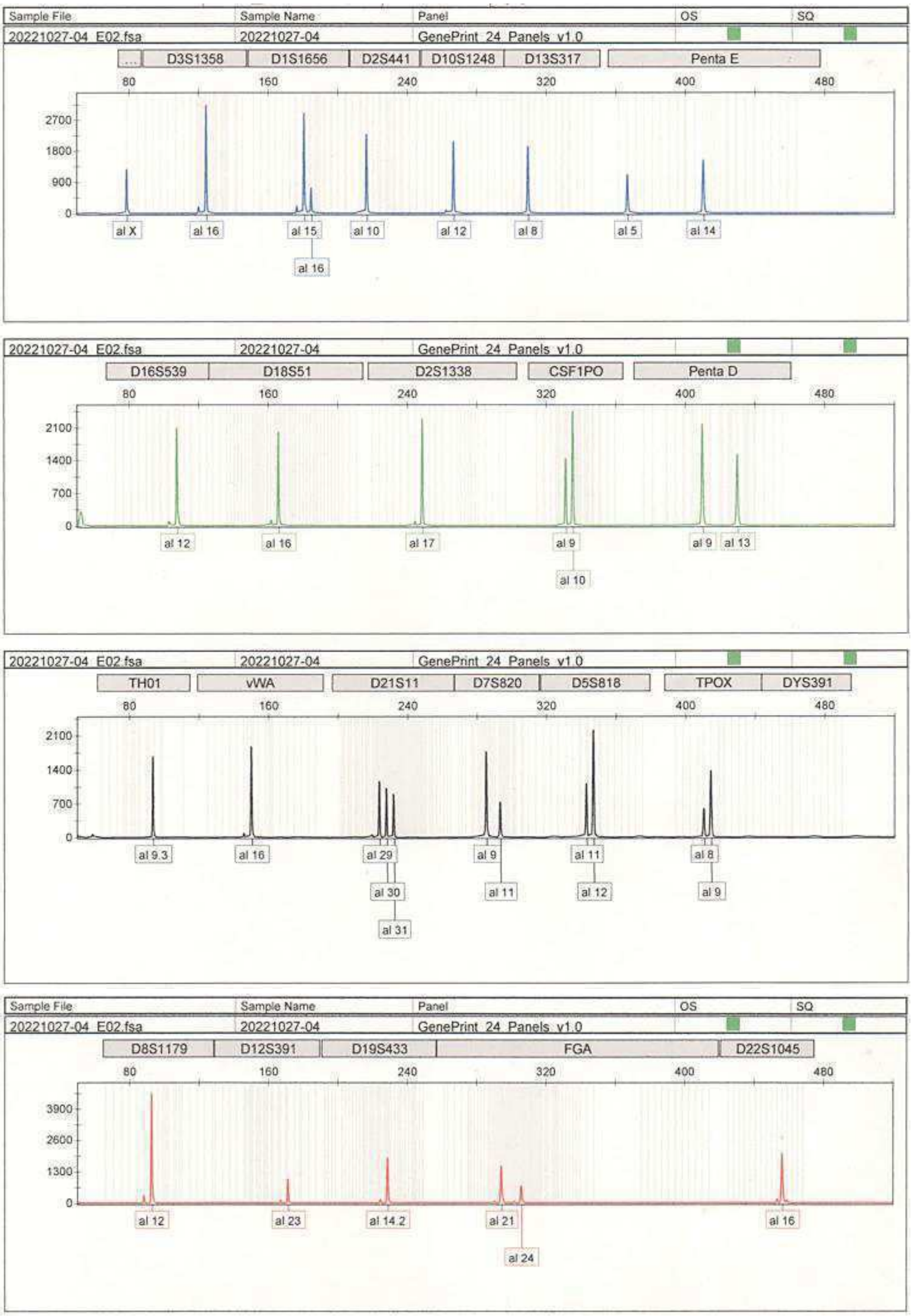

Figure S2

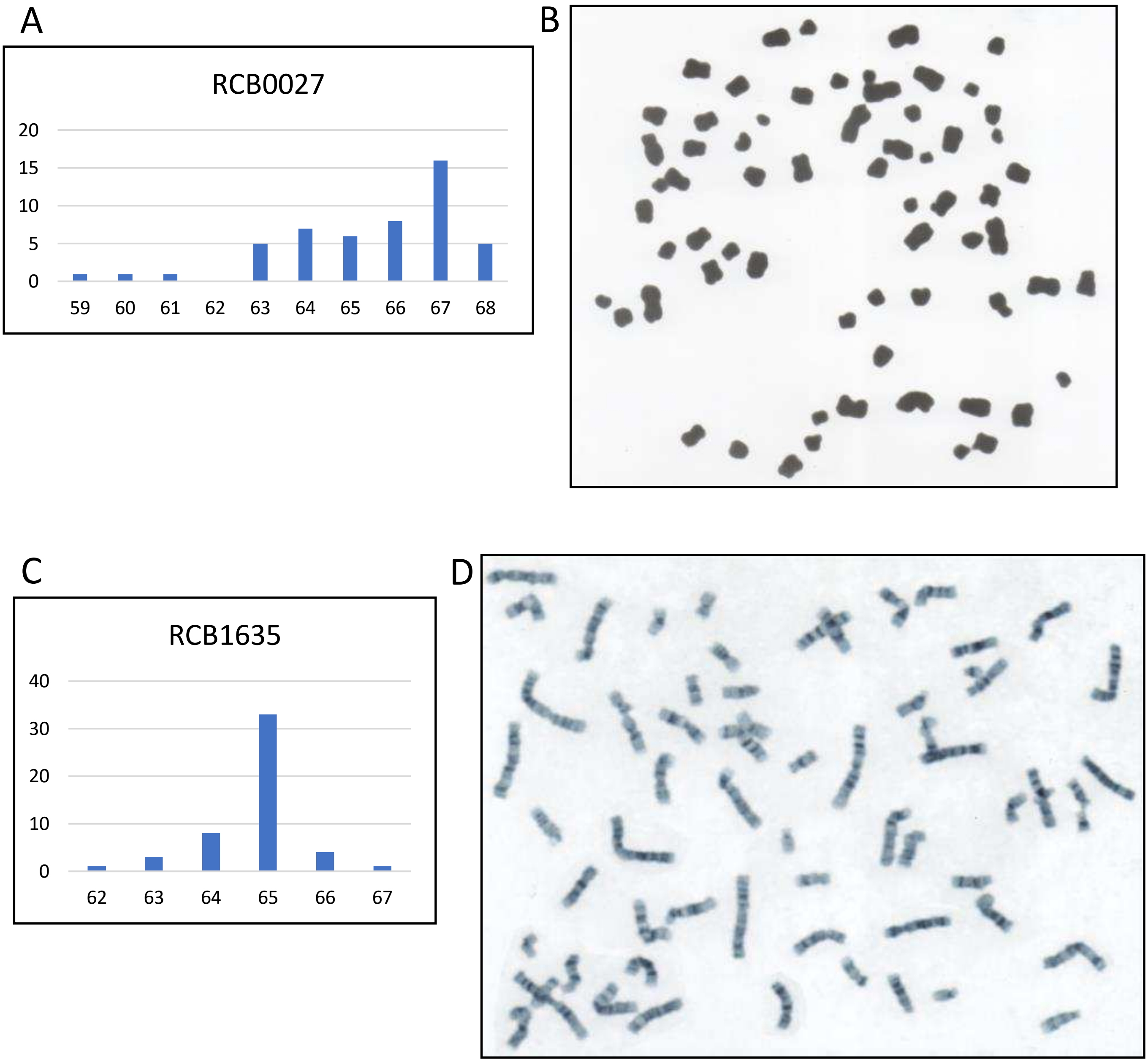

Chromosome analysis of RCB0027(A,B) and RCB1635(C,D). A. The modal chromosome number of RCB0027 is 67, as determined by counting 50 cells. B. An example of a the Giemsa-stained chromosomes from RCB0027, consisting of 65 chromosomes. C. The modal chromosome number of RCB1635 is 65, as determined by counting 50 cells. D. An example of a G-banding chromosomes of RCB1635, consisting of 65 chromosomes.

Figure S3

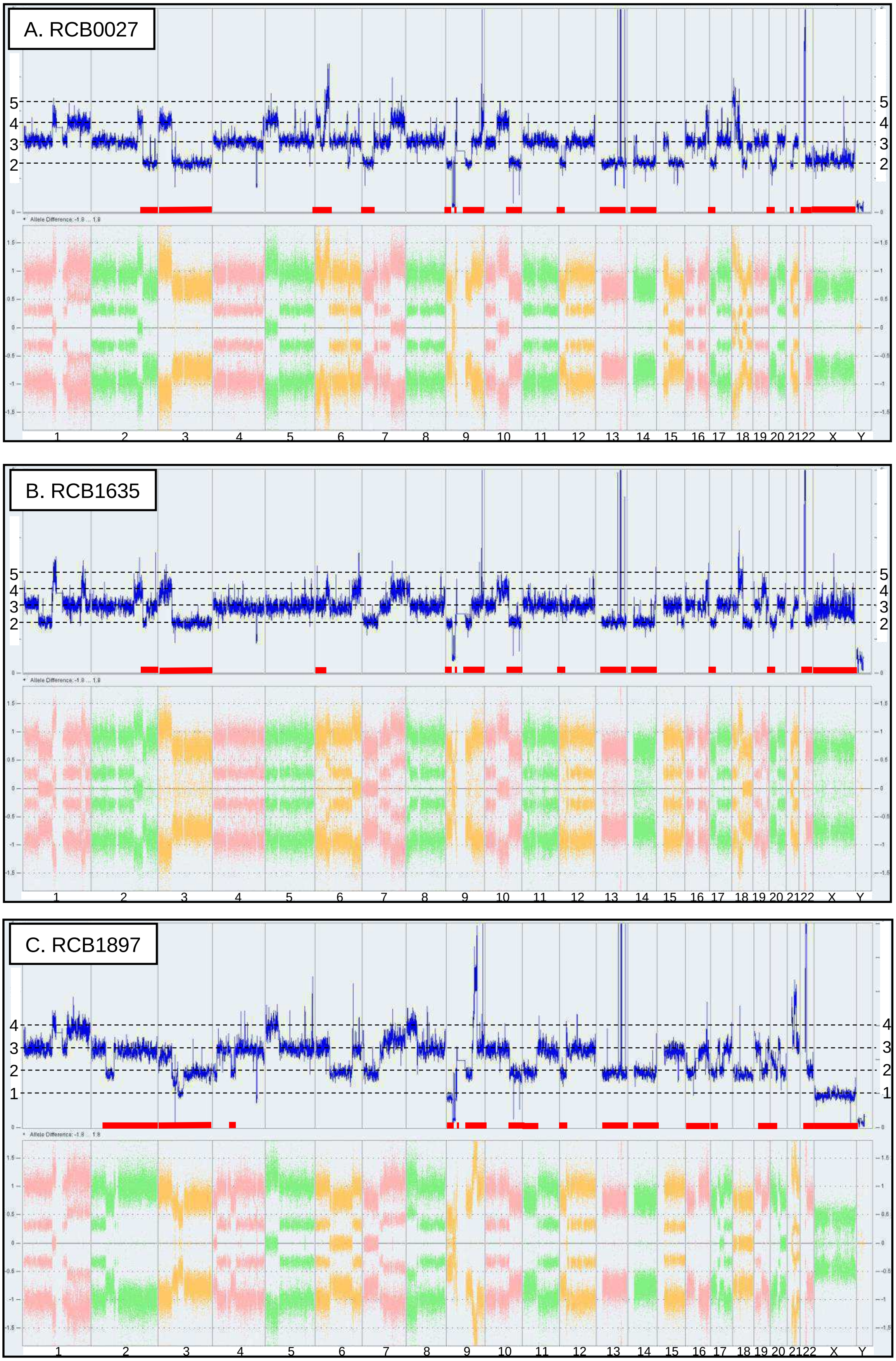

Whole genome profiles obtained from SNP microarray analysis of RCB0027 (A), RCB1635 (B) and RCB1897 (C). The upper diagrams display DNA copy numbers and allele differences across chromosomes. Copy number changes can be observed as either gains or losses, which are indicated by values above or below 2, respectively. Allelic patterns consisting of two peaks correspond to LOH regions.

Figure S4

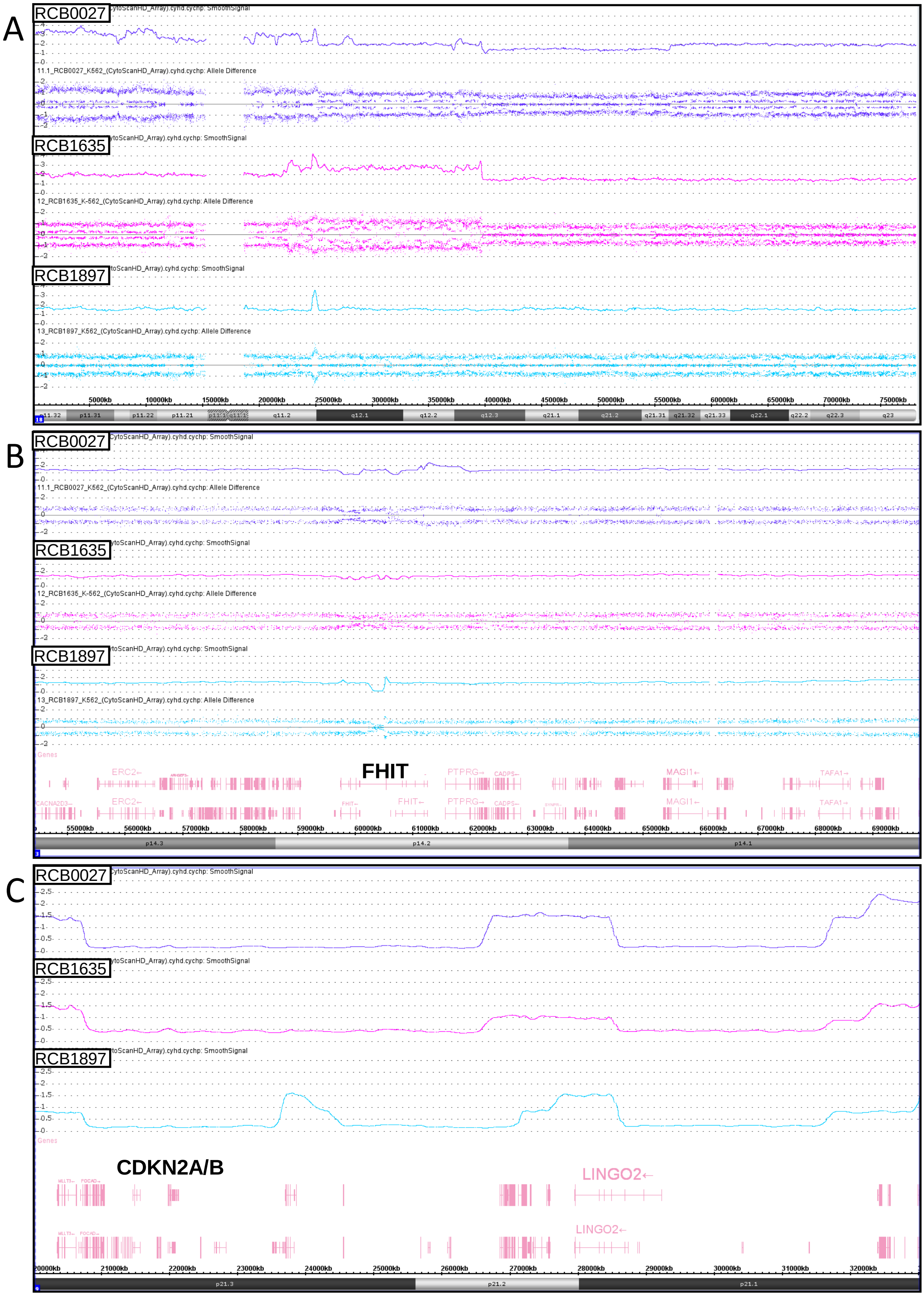

A. Patterns of copy number changes along chromosome 18 indicate differences among the three K-562 cell lines. RCB0027 exhibits several gains and losses along chromosome 18, indicating the occurrence of multiple rearrangements. B. Deletions are detected in the FHIT region at 3p14.2; however, the position differs among the three sublines. C. Deletions at 9p21 result in the homozygous loss of CDKN2A/B.

Figure S4

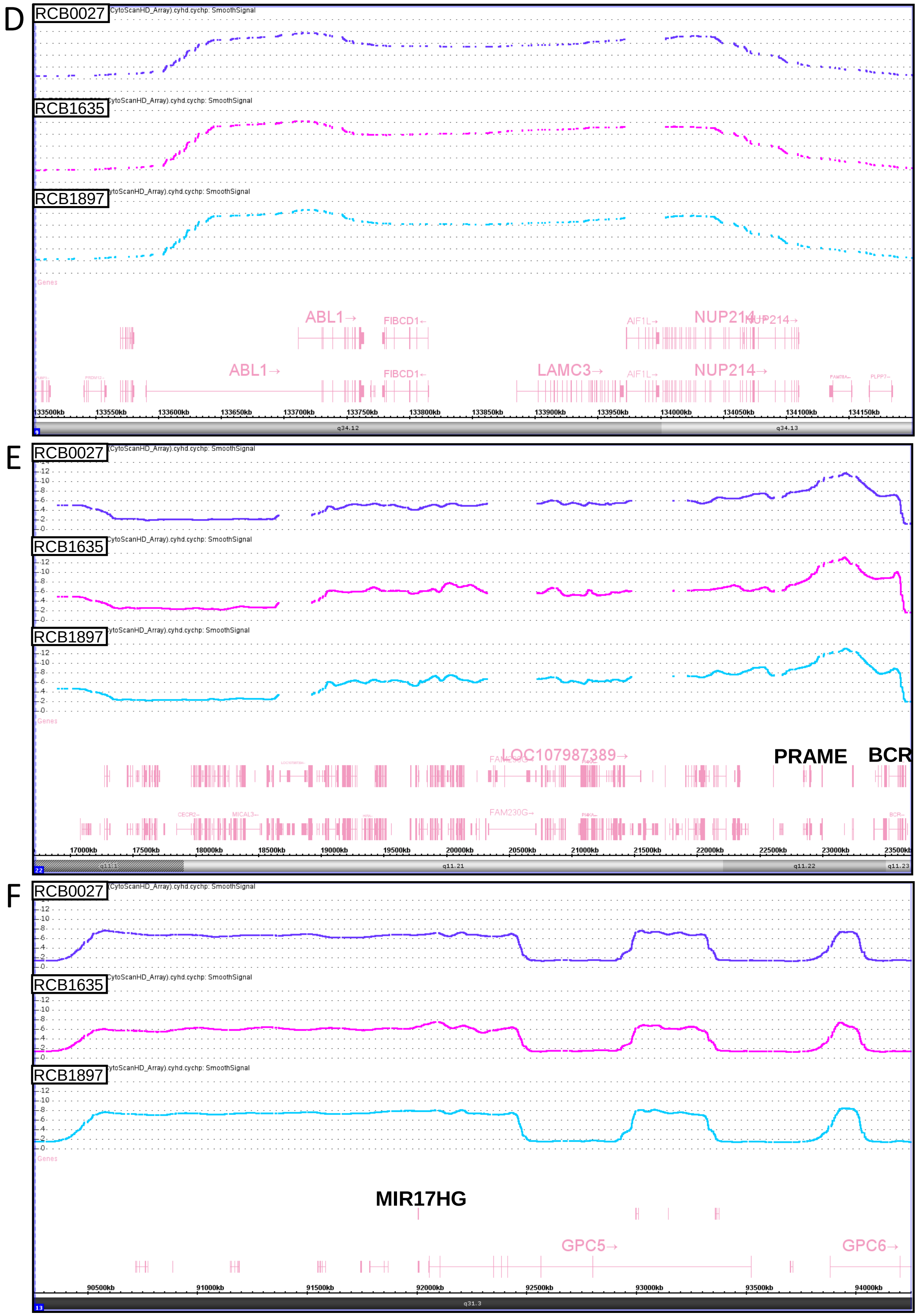

Cryptic changes detected by SNP microarrays show amplifications at 9p34.12 including ABL1 and NUP214 (D), at 22q11 including BCR and PRAME (E), and at 13q31.3 including MIR17HG and GPC5 (F). These profiles are identical across the three sublines, suggesting that the amplifications occurred in the common ancestral K-562 clone.

Figure S4

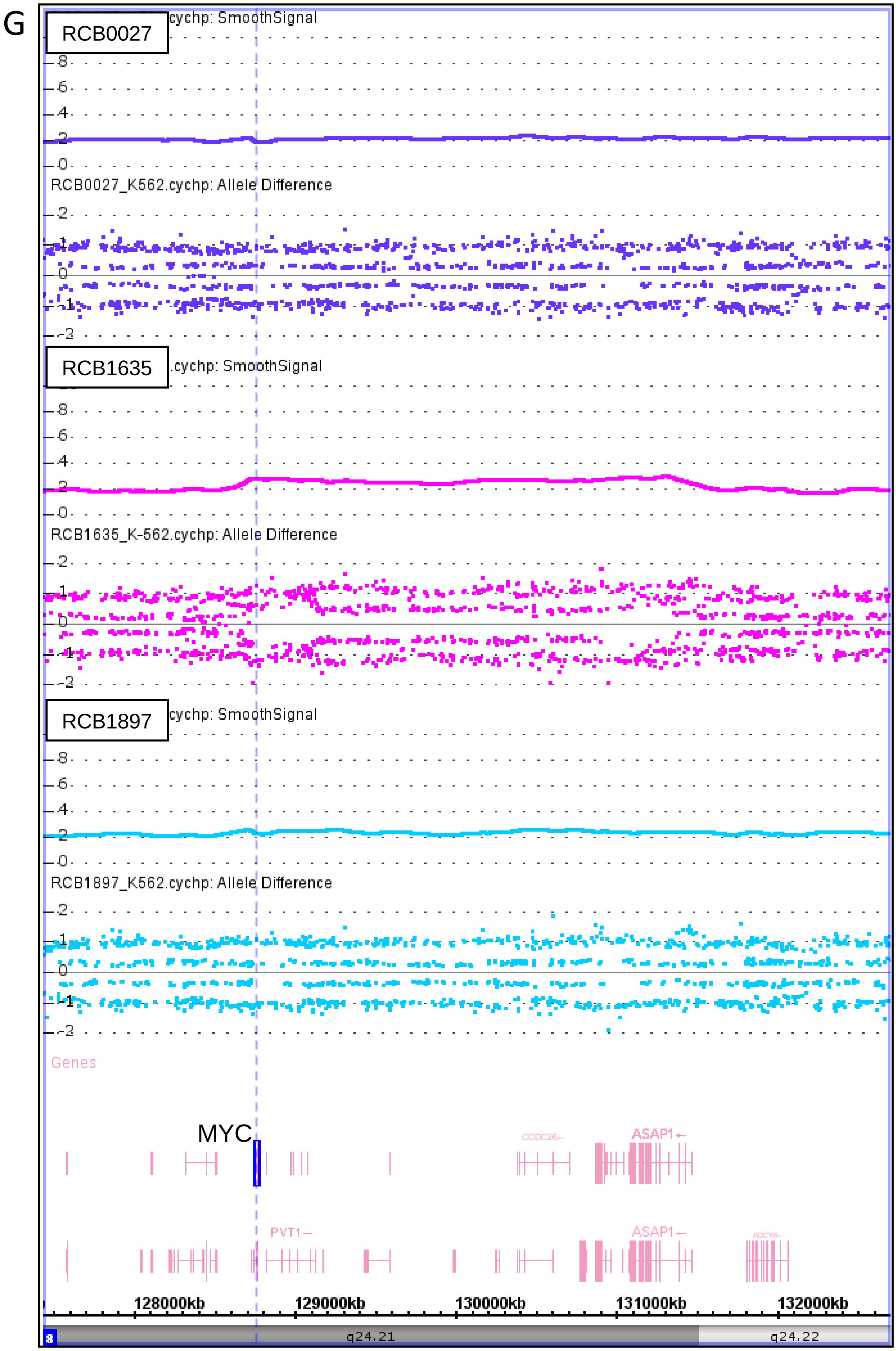

G. A focal gain at 8q24.21 specifically observed in RCB1635. This region includes *MYC*, *PVT1*, and *ASAP1*.

Figure S5

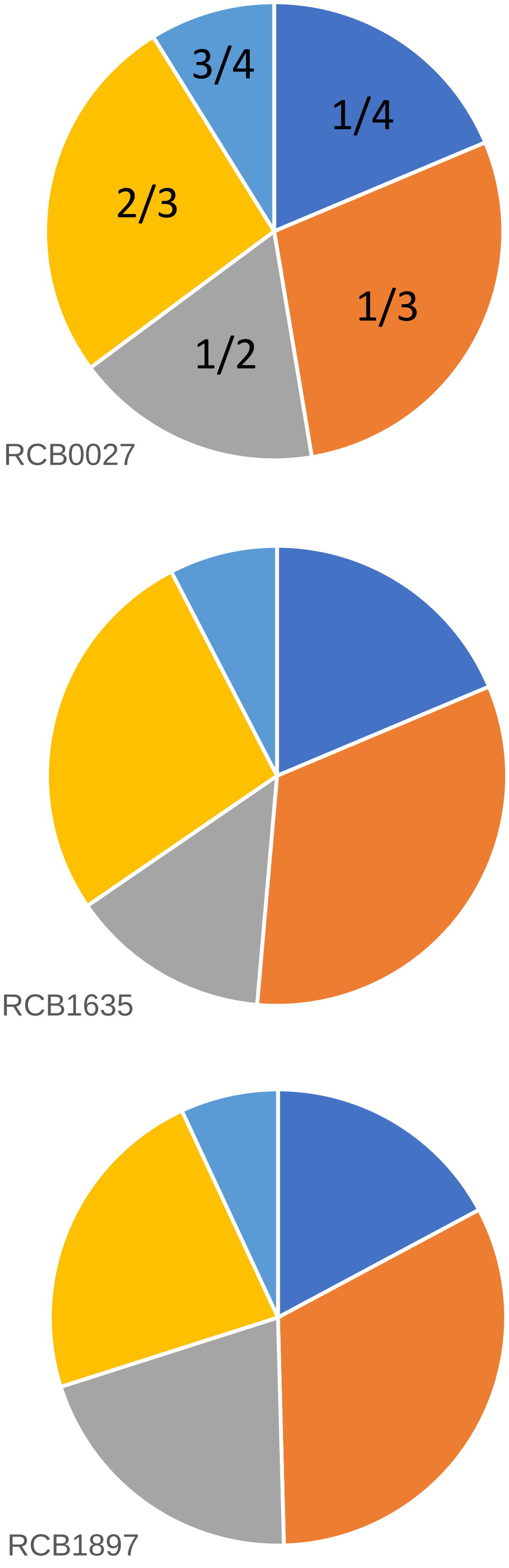

Distribution of variant frequencies. Sequence variants detected by amplicon sequencing using the CCP panel correspond to differences from the hg19 reference data, including SNPs. The variants are sorted based on allelic frequencies according to the five ranges (Table S7). Patterns of the frequencies are similar among the three K-562 sublines, indicating that majority of frequencies are 1/3 and 2/3. This suggests that the genomes of these three sublines consist of triploid ranges.

Figure S6

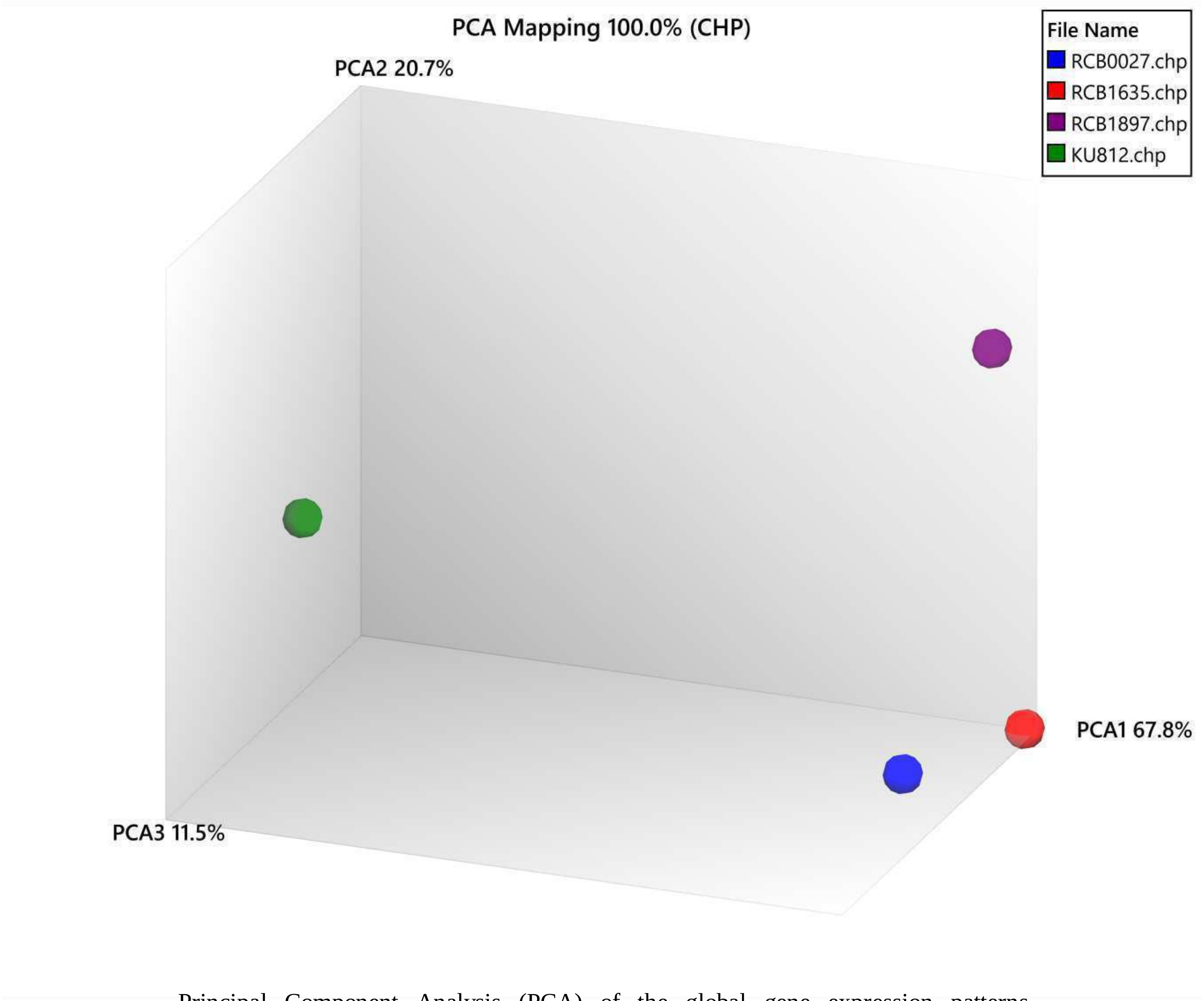

Principal Component Analysis (PCA) of the global gene expression patterns performed on three K-562 sublines and KU812. Based on the three components of PCA1 (67.8%), PCA2 (20.7%) and PCA3 (11.5%) variances, the three K-562 sublines were plotted at a similar level of PCA1 and were clearly distinct from KU812. This indicates that KU812 can be used as an outgroup for the analysis of K-562 sublines.

Figure S7A

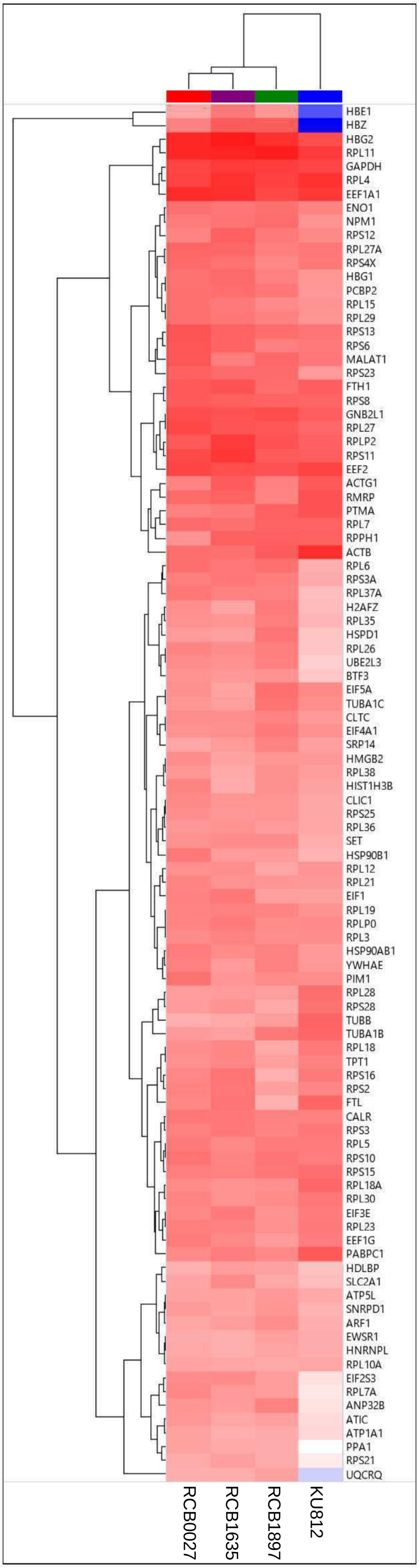

Top 100 genes with high expression levels that are common to the three K-562 sublines. Compared to KU812, K-562 cells can be distinguished by their expression levels of HBE1 and HBZ.

Figure S7B

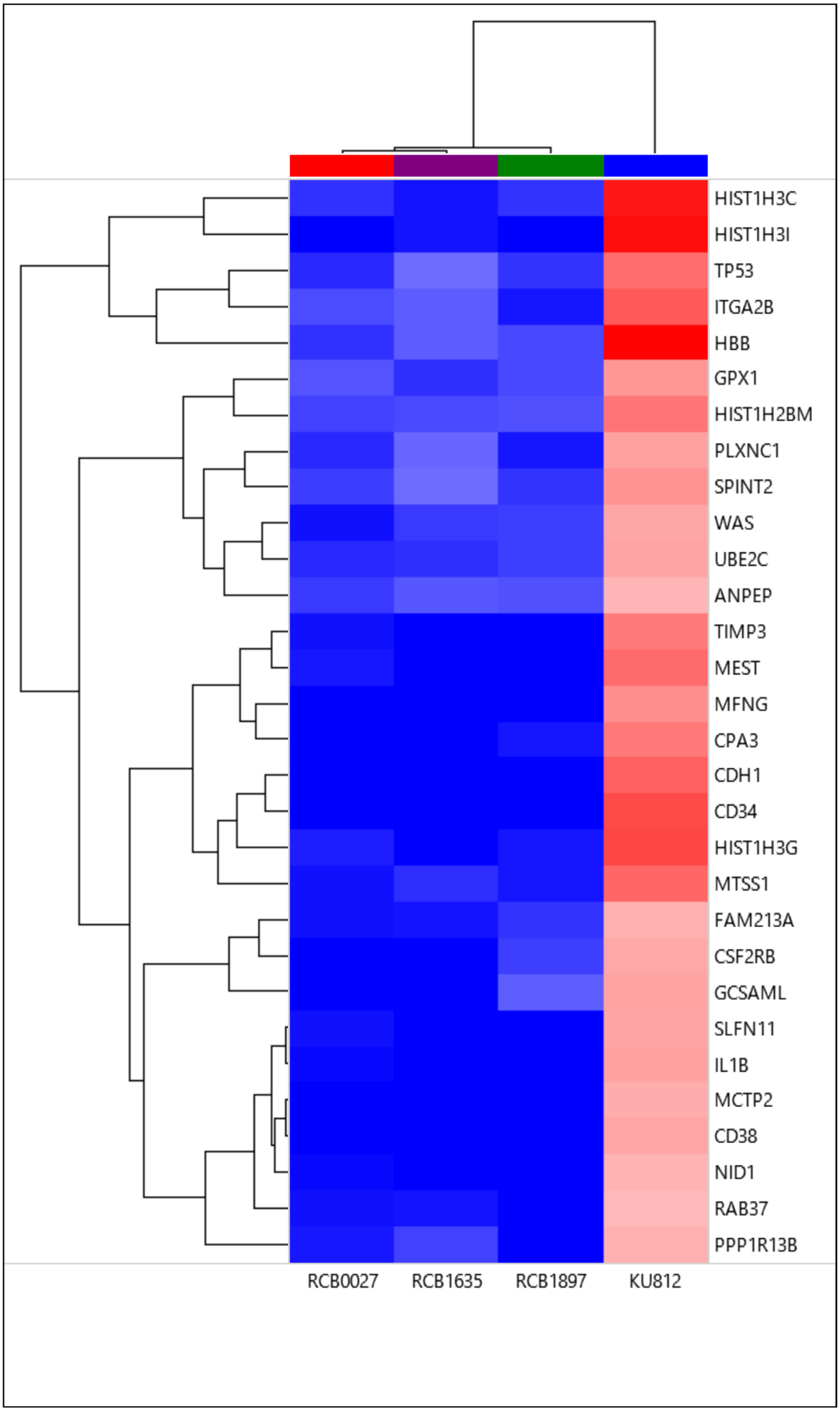

DEGs between three K-562 sublines and KU812. HBB is not expressed in any of the three K-562 sublines, but it is expressed in KU812.

Figure S7CD

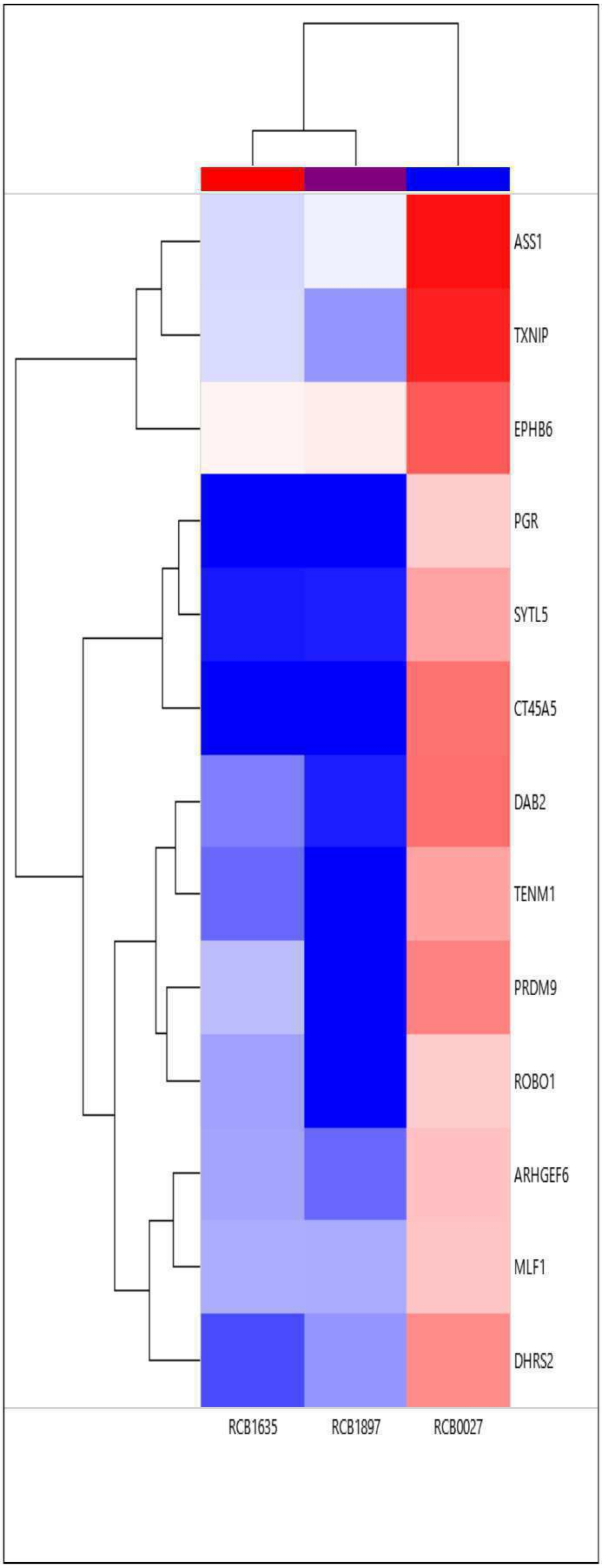

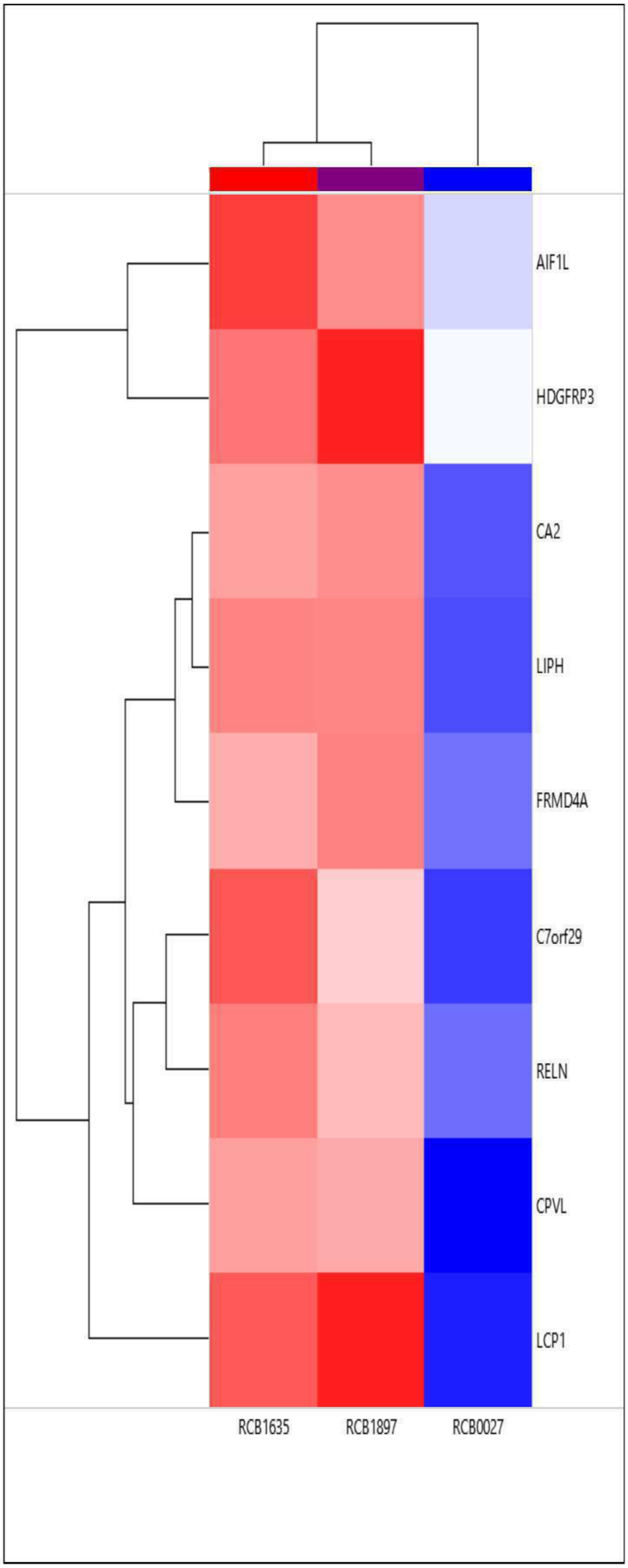

DEGs between RCB0027 and the other two K-562 sublines, showing
13 upregulated genes and 9 downregulated genes, listed in Table S8CD.

Figure S7EF

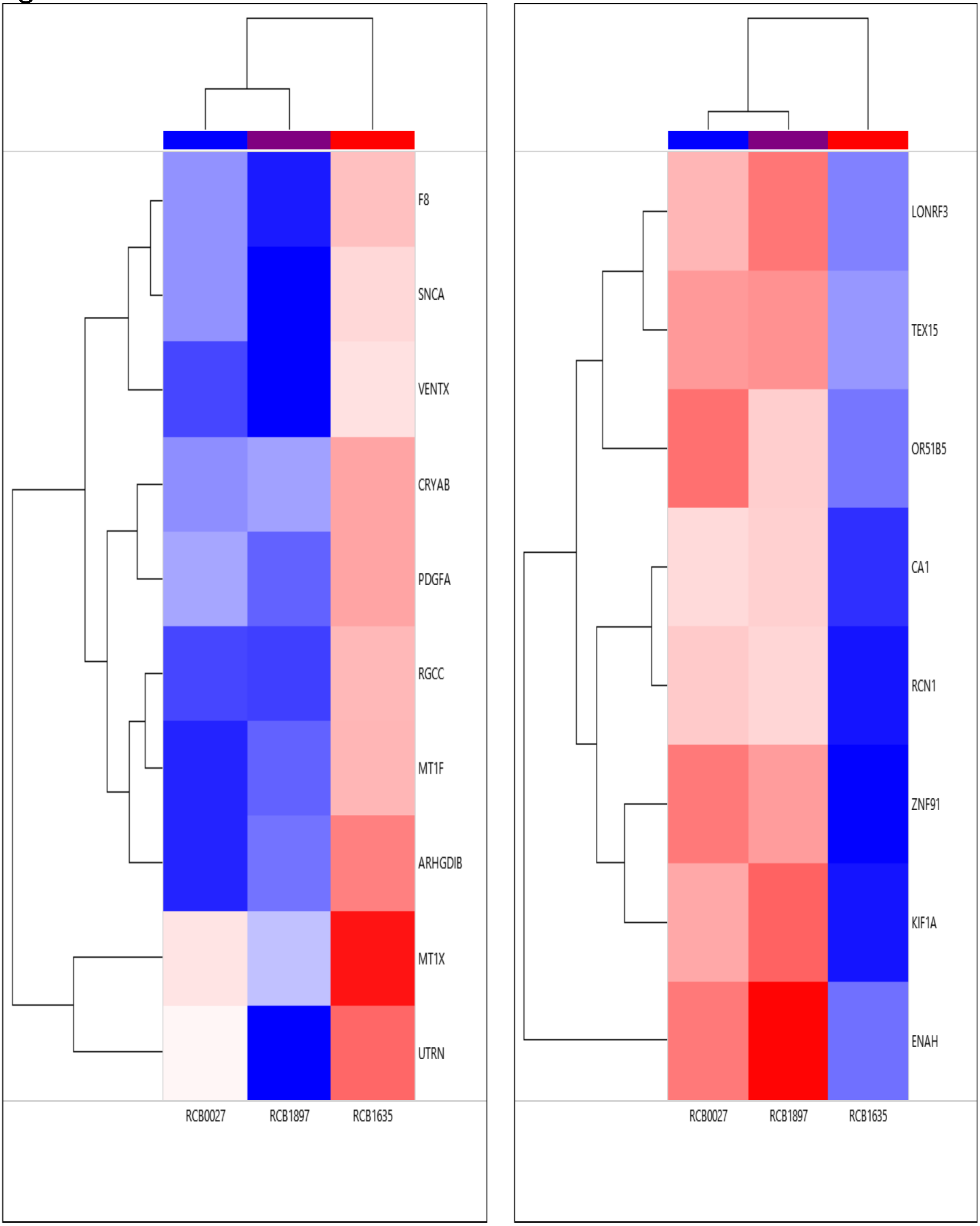

DEGs between RCB1635 and the other two K-562 sublines, showing
10 upregulated genes and 8 downregulated genes, listed in Table S8EF.

Figure S7GH

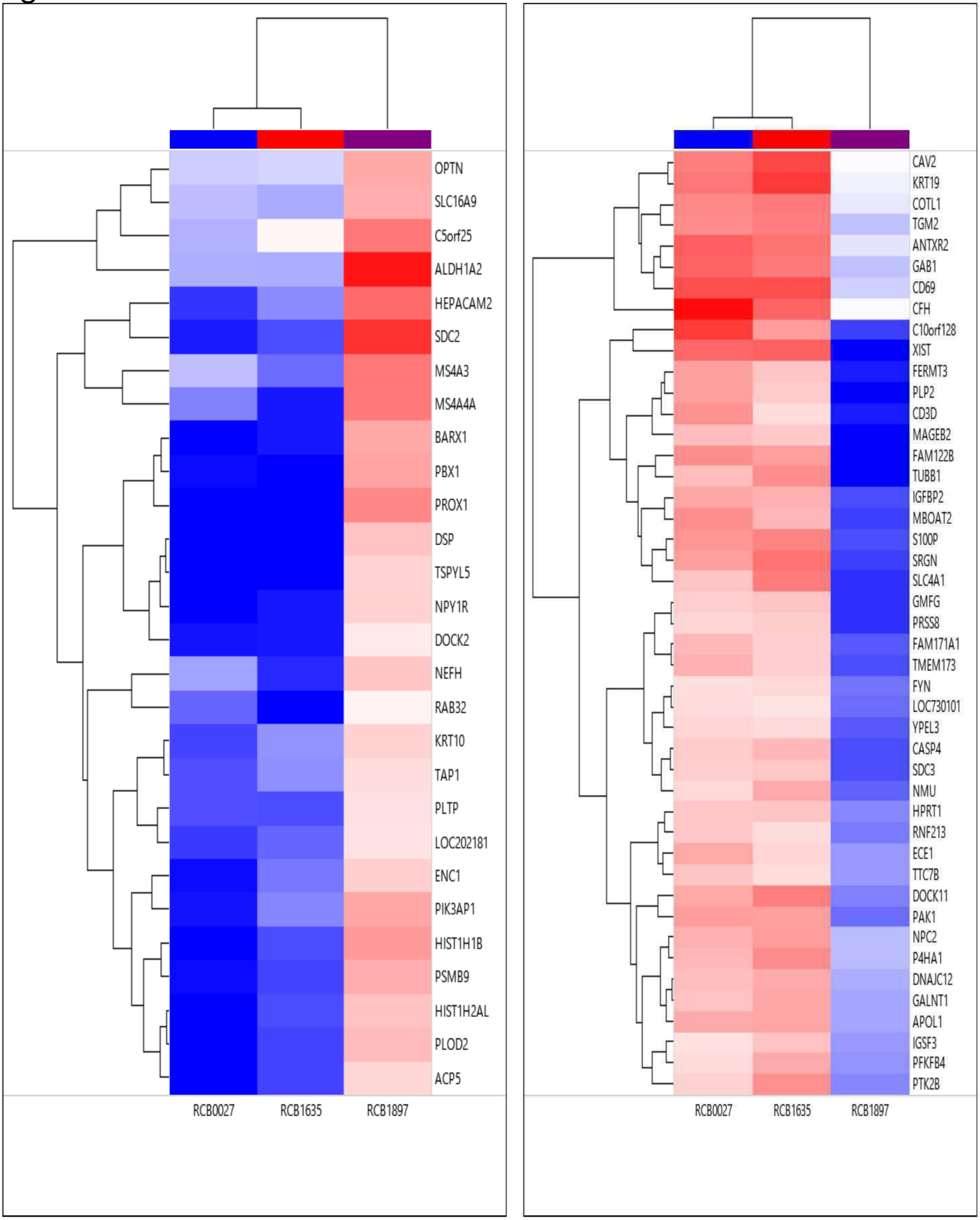

DEGs between RCB1897 and the other two K-562 sublines, showing 28 upregulated genes and 45 downregulated genes, listed in Table S8GH.

Figure S7I

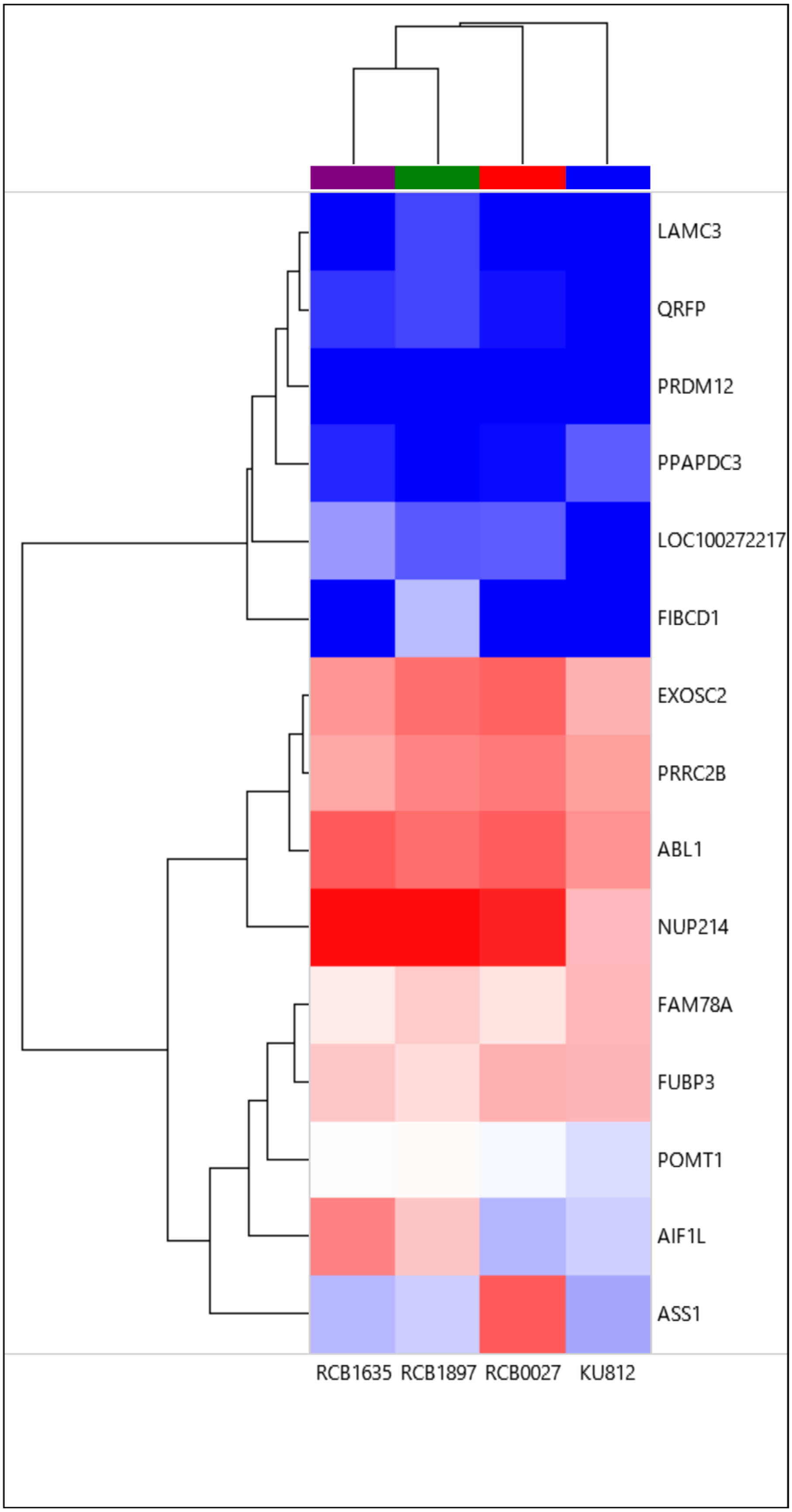

Gene expression patterns in the 9q34.1 region. AIF1L, which is highly expressed in RCB1635 and RCB1897, is also amplified ajacent to NUP214.

Figure S7J

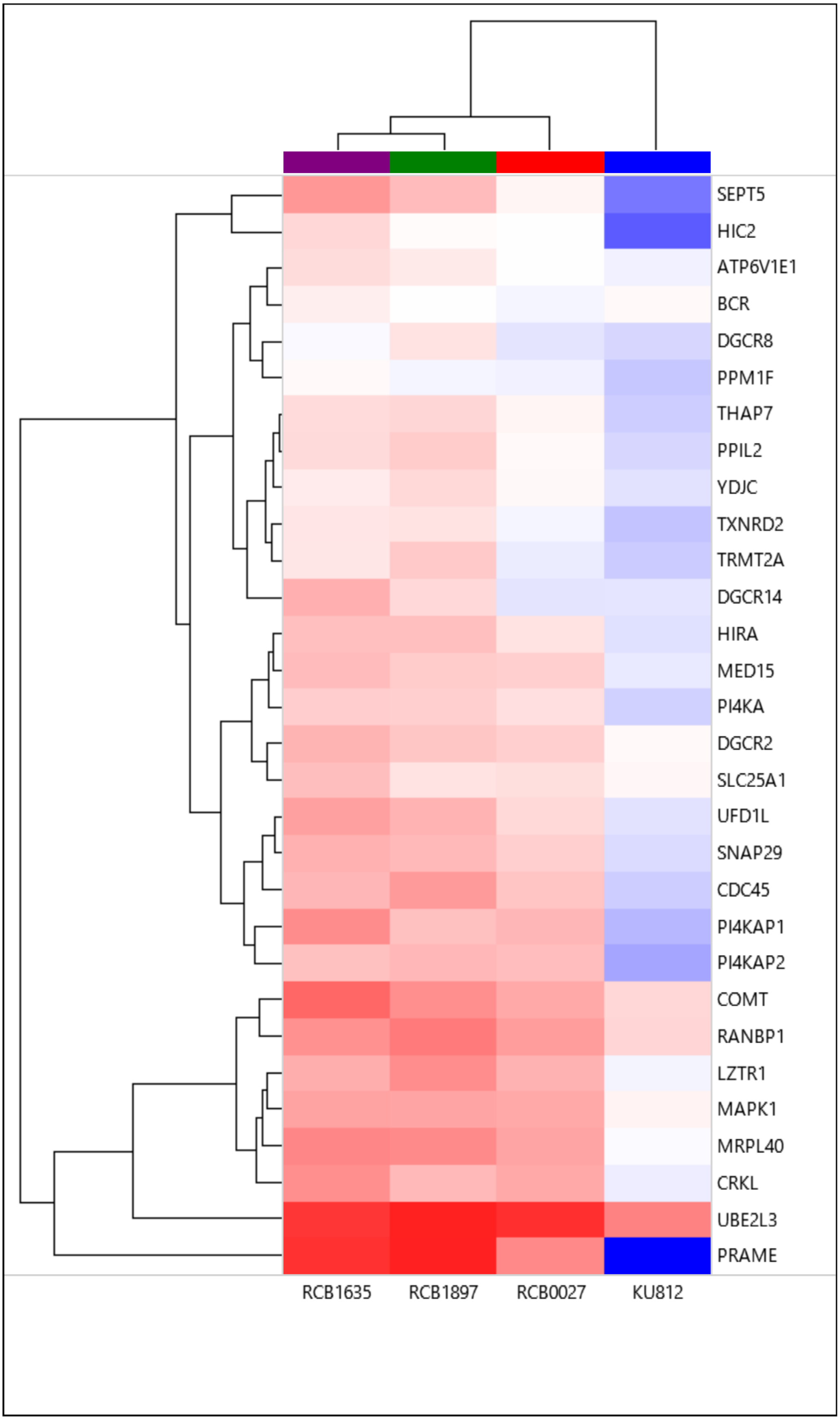

Gene expression pattern in the 22q11.2 region. The PRAME gene, which is amplified with BCR, exhibits high expression levels in the three K-562 sublines, but not in KU812.

Figure S7K

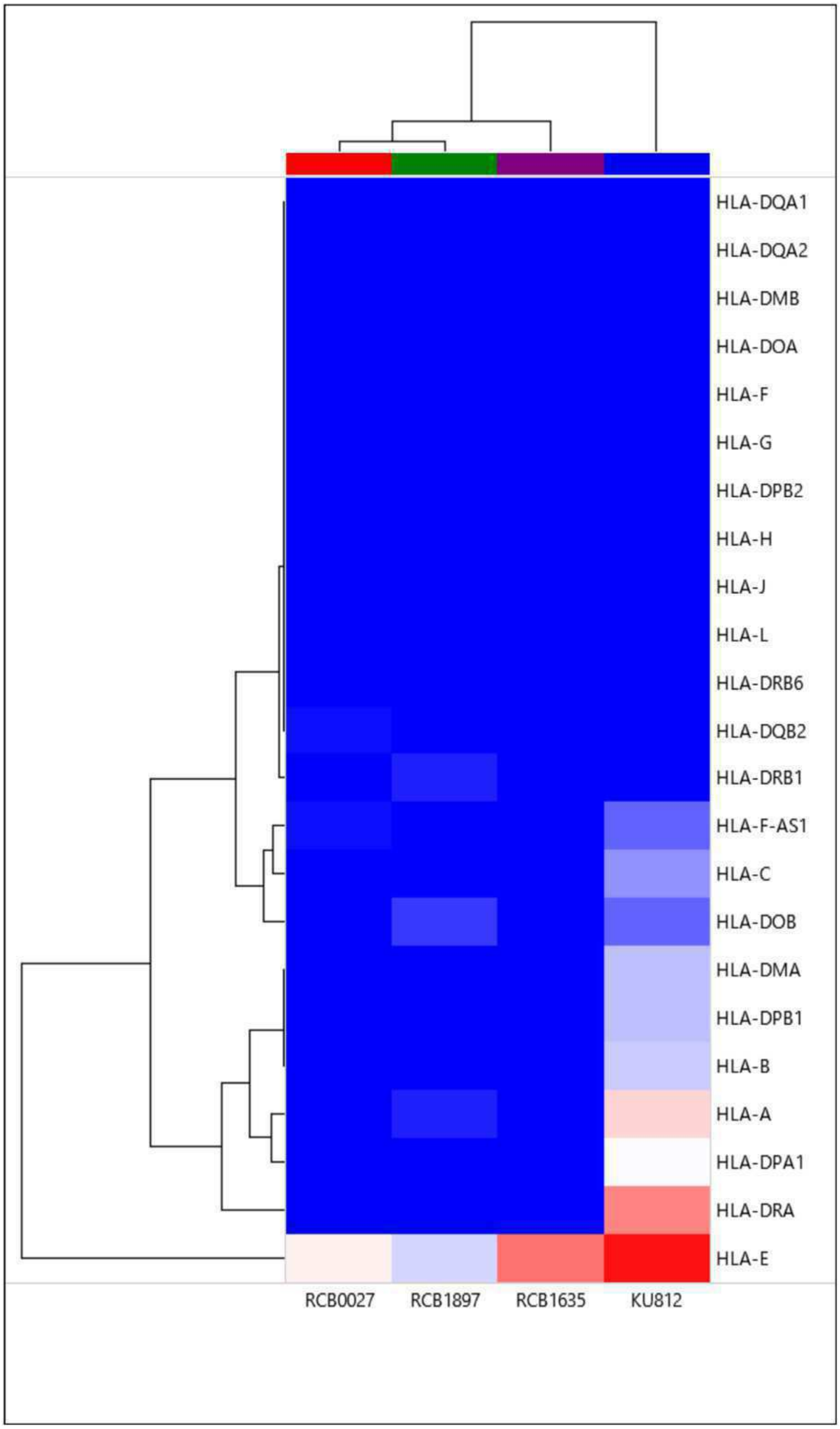

Among the HLA genes, only HLA-E expression was detected in all three sublines, while the other genes were not detected.

Figure S8

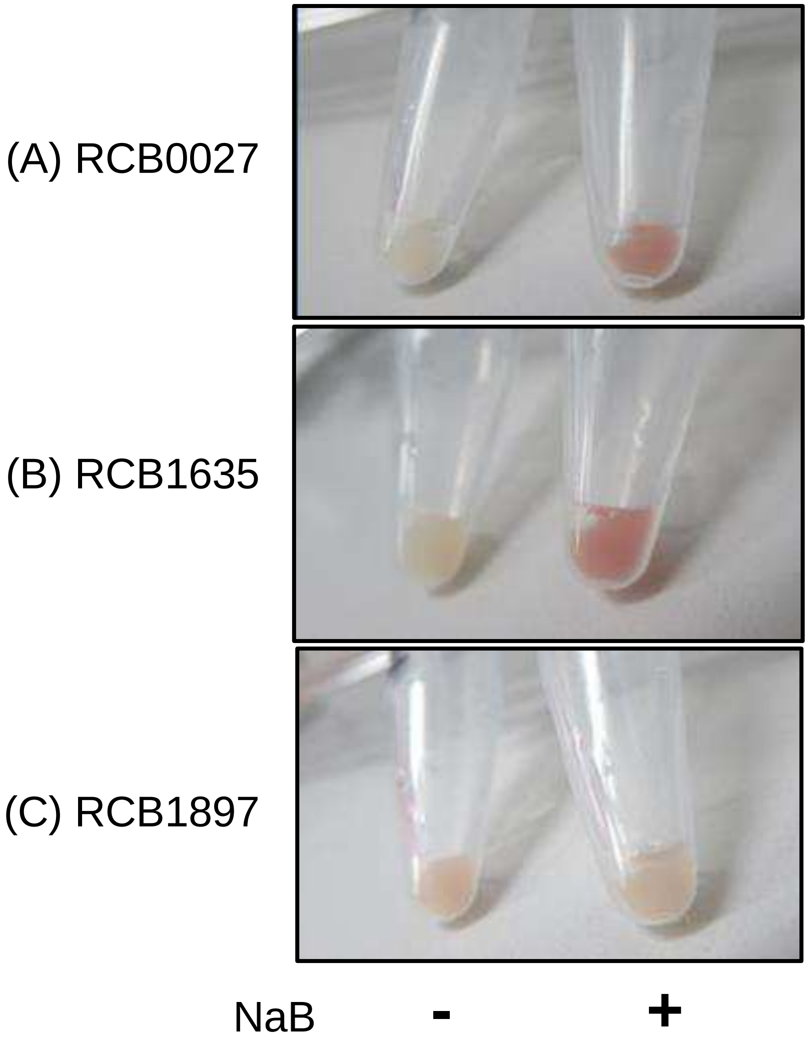

Hemoglobin synthesis induced by sodium butyrate (NaB). Cells were collected after treating them with NaB for 96 hours. The cell pellets showed red color in the NaB-treated RCB0027(A) and RCB1635(B) samples, indicating that hemoglobin was synthesized. However, hemoglobin synthesis was not observed in RCB1897(C).
